# Root System Architecture from Coupling Cell Shape to Auxin Transport

**DOI:** 10.1371/journal.pbio.0060307

**Published:** 2008-12-16

**Authors:** Marta Laskowski, Verônica A Grieneisen, Hugo Hofhuis, Colette A. ten Hove, Paulien Hogeweg, Athanasius F. M Marée, Ben Scheres

**Affiliations:** 1 Department of Biology, Oberlin College, Oberlin, Ohio, United States of America; 2 Molecular Genetics Group, Department of Biology, Utrecht University, Utrecht, The Netherlands; 3 Theoretical Biology Group, Department of Biology, Utrecht University, Utrecht, The Netherlands; Max Planck Institute for Developmental Biology, Germany

## Abstract

Lateral organ position along roots and shoots largely determines plant architecture, and depends on auxin distribution patterns. Determination of the underlying patterning mechanisms has hitherto been complicated because they operate during growth and division. Here, we show by experiments and computational modeling that curvature of the *Arabidopsis* root influences cell sizes, which, together with tissue properties that determine auxin transport, induces higher auxin levels in the pericycle cells on the outside of the curve. The abundance and position of the auxin transporters restricts this response to the zone competent for lateral root formation. The auxin import facilitator, AUX1, is up-regulated by auxin, resulting in additional local auxin import, thus creating a new auxin maximum that triggers organ formation. Longitudinal spacing of lateral roots is modulated by PIN proteins that promote auxin efflux, and *pin2,3,7* triple mutants show impaired lateral inhibition. Thus, lateral root patterning combines a trigger, such as cell size difference due to bending, with a self-organizing system that mediates alterations in auxin transport.

## Author Summary

Plant architecture is determined by where shoots or roots form along the main axis, but the mechanism responsible for lateral root initiation has long puzzled biologists. Here, we show that stretching root cells initiates changes in hormone transport, leading to lateral root initiation in plants, thereby solving a 120-year-old mystery: the mechanism of lateral root initiation. Our data reveal that physical tissue deformation is sufficient to induce chemical changes that unleash biological responses leading to new organ formation. When roots bend, concentrations of the plant hormone auxin increase along the outside of the bend. A complex auxin flux pattern is generated that further enhances auxin levels through localized reflux loops. Auxin importers—AUX1—and efflux carriers—PIN proteins—are known to be regulated by auxin. AUX1 up-regulation enhances the auxin maxima that specify the lateral root founder cells at the bend, while PIN down-regulation modulates the lateral spacing of the roots along the main root axis. This study shows that the biological regulation behind pattern formation can be a result of entangled hierarchies, explaining both the inner/outer spacing, lateral inhibition, and dynamics of lateral root initiation.

## Abstract 3

Experimental data and computer modeling show that lateral root positioning can be controlled by the physical stimulus of root curvature, which triggers self-organizing alterations in auxin transport.

## Introduction

**Figure 1:**
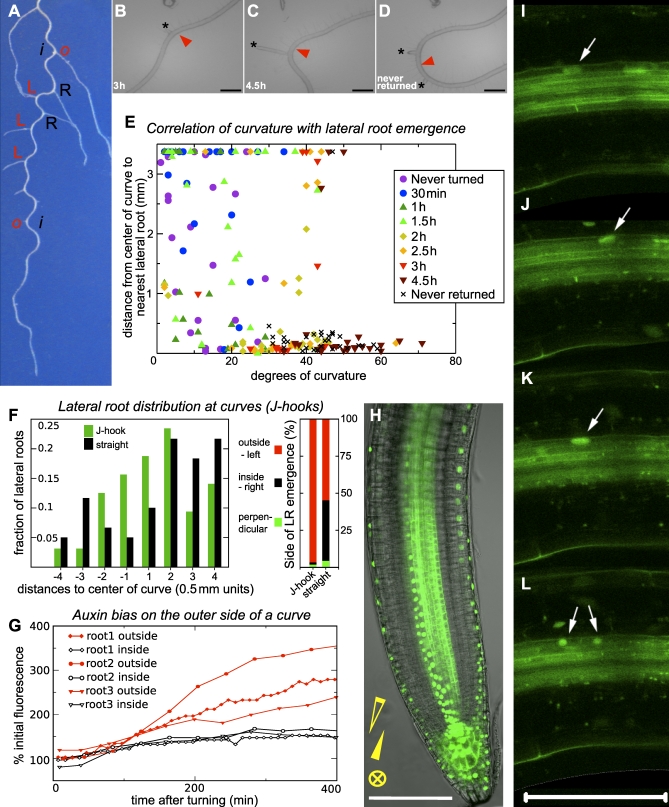
Lateral Root Initiation Is Induced by Root Curvature (A) Lateral roots are formed on the outside of curves in an alternating left/right rhythm (*o* indicates the outside, and *i* the inside of curve; L indicates left, and R right, relative to the main axis of the root). (B–D) Examples of root curvature resulting from gravitropic stimulation of different time intervals (B) 3 h; (C) 4.5 h; (D) never returned. Black asterisk (*) indicates the position of the emerged lateral root; red arrowhead indicates the center of curve. Scale bar represents 500 μm. (E) Lateral root formation correlates with the degree of root curvature resulting from gravitropic stimulation over various amounts of time. Symbols indicate time in the inverted position. Distance from the center of the curve to the nearest emerged lateral root is reported. (F) Lateral root initiation is induced in manually curved roots. Left: location along the curve where lateral roots form is reported alongside the comparable position(s) for straight roots. Center of curve is defined as zero, and negative values are closer to the root tip, distal to the center of the curve. The curve was made 0.5 cm from the root tip. Right: percentage of lateral roots forming on each side of the main root. Sides are defined as inside and outside for curved roots, and left and right for straight roots, as shown in Figure 1A. (G) DR5::GFP accumulates asymmetrically in the stele of manually curved roots. Solid red symbols indicate the outer half of the stele; open black symbols indicate the inner half. (H) Root curvature due to gravitropic response results in inverse asymmetric auxin distributions in the primary root MZ. DR5::vYFP (nuclear), PIN7:GFP (ER). Open arrowhead indicates the gravity vector during initial growth; the solid arrowhead indicates the gravity vector during the period of inversion; and the circled cross indicates the gravity vector directed into the plane during imaging. Scale bar represents 100 μm. (I–L) Auxin response is enhanced locally in the pericycle and adjacent endodermal cell at a curve prior to the asymmetric cell division. Fluorescent markers as in (H). (I) 300 min, (J) 110 min, and (K) 10 min before, and (L) 10 min after the pericycle cell division. Arrows mark the location of the dividing nucleus. Scale bar represents 100 μm.

Developmental biologists often conceptualize patterning mechanisms in uniform fields of cells, but in reality, positional information may be generated and translated in dynamic circumstances in which cells divide, grow, and change shape. These circumstances allow for unexpected feedbacks, making analysis a challenge. Rhizotaxis, the arrangement of lateral organs along plant roots, is a good example of a patterning process that occurs in a dynamic context. The mechanisms that regulate rhizotaxis have long remained a mystery, although the origin of lateral roots was described as early as 1888 [1]. In *Arabidopsis*, lateral roots arise from two files of pericycle cells that lie adjacent to the protoxylem [2], and the pattern of emerged lateral roots can be described in terms of the longitudinal spacing along a file and the left/right component (Figure 1A). The longitudinal pattern is variable and cannot be explained by mechanisms that require a fixed amount of time, distance, or number of pericycle cells between initiations [3]. There is, however, a strong tendency for lateral roots to arise on the outside, i.e., convex side, of the curve [4]. This tendency correlates with above-average auxin response at the proximal end of the meristematic zone (MZ) well before the first asymmetric divisions [5]. Lateral root formation can be induced by global increases in auxin content [6], and more specifically, by local activation of auxin synthesis in pericycle cells [7]. Mutations that render plants less sensitive to auxin reduce lateral root numbers [8,9]. Additionally, chemical or genetic inhibition of auxin transport can decrease lateral root density [10–12]. These observations indicate that lateral root formation is influenced by auxin, but they do not reveal the underlying mechanism. Here, we combine experimental and multilevel modeling approaches to unravel the molecular and biophysical mechanism that regulates rhizotactic patterning.

## Results

### The Formation of a Curve Induces Lateral Root Initiation and Explains Left/Right Positioning

Consistent with prior reports of a correlation between lateral root formation and the presence of curves, we observed that a waving growth pattern increases the average density of emerged lateral roots (1.6 and 1.8 vs. 2.5 and 2.6 emerged lateral roots/centimeter for “straight” grown vs. waving roots in each of two experiments, where by “straight,” we refer to vertically grown roots that curve less than those grown on slanted agar).

To determine how the degree of curvature impacts lateral root formation, we inverted *Arabidopsis* seedlings for variable lengths of time before returning them to an upright position (Figure 1B–1D). The degree of curvature and the probability that a lateral root is located on that curve both increased with time (Figure 1E). Strikingly, roots with curves of more than 45° all developed associated lateral roots, indicating a strong correlation between the presence of a sharp curve and lateral root induction (Figure 1E and Protocol S1: Figure S1A and S1B for details). To determine whether lateral root positioning is influenced by the mechanisms that produce undulating root growth or by curvature itself, we bent roots in the shape of a J, 0.5 cm from the root tip, in regions where cells are fully differentiated. Lateral root formation was examined in the curved region and around the comparable position that was marked on straight-grown control roots. Lateral roots arose along the entire length of the curve, effectively extending the zone of lateral root formation closer toward the root tip (Figure 1F, left). The average distance from the center of the curve to the closest lateral root is less than from the control mark to a lateral, demonstrating that curves promote lateral root formation. Furthermore, formation of new lateral roots was strongly biased toward the outside of the curve (Figure 1F, right). We conclude that curvature of a root by itself focuses lateral root density along the longitudinal axis and establishes the left/right position of lateral roots. Roots that curve all along the longitudinal axis do not display lateral roots in the MZ and the elongation zone (EZ), indicating that only the differentiation zone (DZ) responds to curvature with lateral root formation.

To investigate whether curving roots in the DZ induces changes in auxin distribution, roots expressing the auxin response marker DR5::GFP [13] were manually curved and subjected to dynamic confocal imaging. Straight roots reveal strong auxin response maxima in the MZ [14] and a weaker auxin response in the vasculature of the DZ. When a curve was introduced into the DZ, fluorescence initially increased more or less symmetrically across the vasculature, but about 2 h later, the auxin response pattern became biased towards the outside of the curve (Figure 1G and Protocol S1: Figure S1C for details).

To facilitate dynamic analysis of lateral root formation, we developed a system for inducing lateral roots within a narrow region that could be imaged with a confocal microscope. Six-day old plants were turned 180°, left for 4 h, and then placed horizontally under the microscope. Consistent with expectation, plants carrying DR5::venusYFP, a nuclear-targeted fluorescent marker noted for its high intensity and short maturation time [15], show increased levels of auxin response in epidermal cells of the MZ along the inside of the curving root where elongation is inhibited [16,17] (Figure 1H).

However, the situation in a curved region of the DZ is quite different. There, the highest level of auxin response is found in mature vasculature, particularly on the outside of the curve, where lateral roots form (Figure 1G). This is in accordance with the results of our manual curvature experiments. In the pericycle, increased auxin response was first observed about 190 min before the first asymmetric division of the founder cells, then rapidly escalated, far surpassing the level of auxin response in all of the surrounding cells (Figure 1I–1L; Video S1). The endodermal cell located adjacent to the founder pericycle cells underwent a smaller and transient increase in auxin response. In previous studies, Stage I primordia that formed after gravistimulation appeared 3 h after strong curvature was established [18], which agrees well with our data and implies that curvature leads to a rapid increase in auxin levels.

### Modeling Auxin Fluxes in the Entire Root Indicates Critical Differences between Root Zones

**Figure 2:**
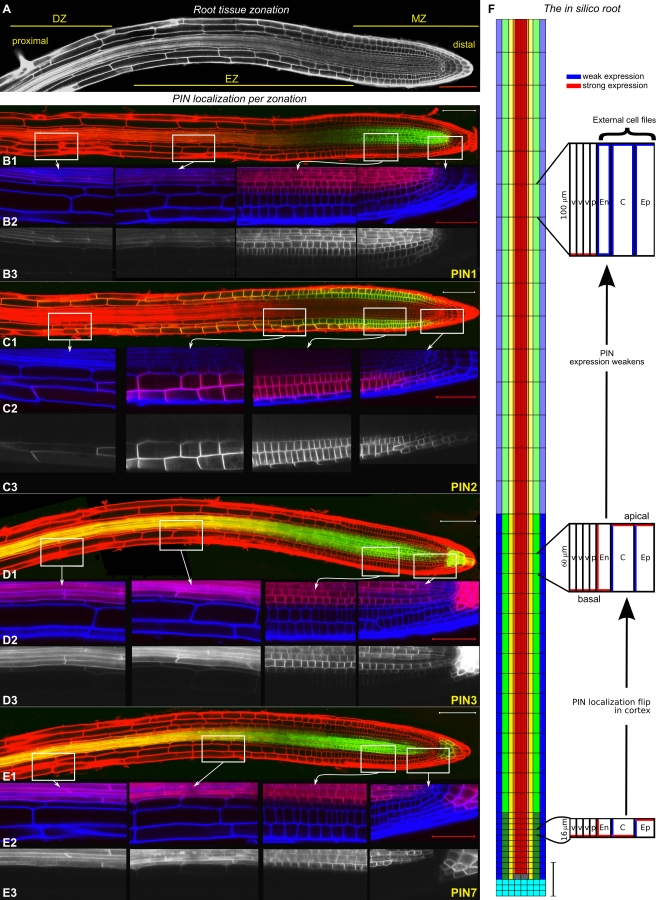
Root and Model Layout (A) Image of a live root, with meristem (MZ), elongation (EZ), and differentiation (DZ) zones indicated. (B–E) PIN expression domains of (B1–B3) PIN1:GFP, (C1–C3) PIN2:GFP, (D1–D3) PIN3:GFP, and (E1–E3) PIN7:GFP. For B1, C1, D1, and E1, the GFP is shown in green and the propidium iodide (PI) stain in red. In B2, C2, D2, and E2 (enlargements of the insets of the DZ, EZ, and MZ in overviews B1, C1, D1, and E1, respectively), the GFP is shown in red and PI channel in blue. In B3, C3, D3, and E3 (enlargements of the insets of the DZ, EZ, and MZ in overviews B1, C1, D1, and E1, respectively), the GFP channel is shown in white. Laser and microscope settings were constant for each marker line. Scale bars represent 100 μm in overviews and 50 μm in enlargements. (F) The in silico root describes the epidermis ([ep]; blue), cortex ([c]; green), endodermis ([en]; yellow), pericycle ([p]; orange), and vasculature ([v]; red). QC (grey) and columella cells (cyan) are only in the distal MZ. Scale bars represent 100 μm. Model cell types are endowed with specific PIN topologies and strengths, which vary by zone. Differences between zones are indicated by changes in color tone. Red indicates strong PIN expression, blue weak. Typical cell lengths vary between zones, as indicated. Cell widths vary between tissue types and are kept constant through the zones. Parameter values are given in Protocol S1: Tables S1–S3 and Text S1.

To understand how root curvature affects lateral root initiation, we developed a model that describes the dynamics of auxin transport through the root. In the model, we consider that auxin can only diffuse freely within cells and in the cell wall, whereas passage of auxin over cell membranes is determined by permeability properties. The efflux and influx permeability values are enhanced by the presence of PIN and AUX1 expression, respectively. The model captures the following basic biophysical characteristics of the system: (1) the overall cell geometries and tissue types; (2) typical lineage- and zone-dependent PIN distributions and expression levels; (3) cell-shape changes due to a mechanical alteration of curvature of the whole organ; and (4) auxin transport itself (see Protocol S1: Tables S1–S3 and Text S1 for a detailed discussion). Given the discrete nature of cells, and the manner in which free diffusion of auxin is interrupted by membranes, auxin dynamics may be strongly influenced by cell shape. The in silico root layout is therefore constructed using typical cell lengths and widths within the MZ, EZ, and DZ. In live roots, cells in the MZ are smaller, but transiently increase length when in the EZ, until reaching a maximally elongated state in the DZ. This is modeled by selecting different characteristic cell lengths for each of the three zones. Differences in width between the external cell files, and the thinner nature of the vascular cells, are also included (Figure 2F and Protocol S1: Text S1 for details). Such models are necessary because simple, intuitive predictions of auxin flow based only on the location of auxin transporters neglect important factors that determine flux patterns within the context of the whole tissue, including the impact of cell size and shape and the fact that the amount of flux through the transport facilitators is determined by the substrate concentrations [19–22].

We performed a systematic analysis of the PIN expression patterns throughout the whole axis of the root (Figure 2A–2E). Our model incorporates the experimentally observed PIN expression topologies, as well as the specific differences between each zone in a schematic manner, by specifying the orientation and distinguishing between strong and weak PIN expression (Figure 2F). Overall PIN expression levels are weaker in the DZ than in the EZ or MZ. Because we first focus on understanding the influence of a root curvature by itself on the auxin dynamics, regulation of PIN or AUX1 expression or localization is initially left out. Using a similar system, we have previously shown that polar auxin transport is sufficient to generate and maintain the auxin maximum at the quiescent center (QC) [22]. PIN localization—in particular the distribution of laterally inward-oriented PINs—is of paramount importance in determining the properties of the root tip [22].

**Figure 3:**
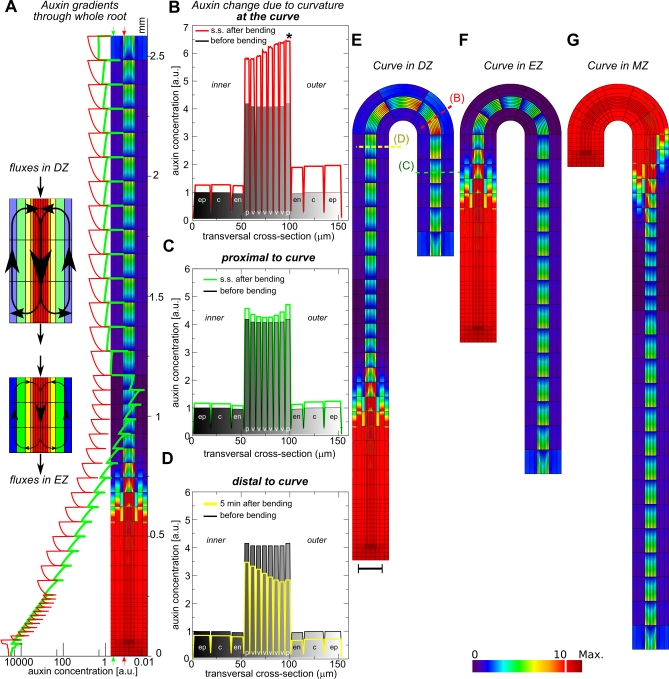
Auxin Gradients and Curvature Effects (A) Steady-state auxin profile through the MZ, EZ, and DZ of a straight root. Longitudinal cross-sections through epidermal and pericycle files reported by green and red lines in graph; in silico root inlaid within the graph, along the *y*-axis indicating distance from root tip, and colors represent auxin concentration levels. Insets on left schematically show increased auxin-reflux loop in the DZ region when compared to a proximal MZ region. (B–D) Transversal auxin profile showing cross-sections through an unbent root (black and shaded) compared with those of a bent root (E), at different locations: at the curve (B), at steady state (s.s.), revealing strong outward concentrations bias; proximal to (above) the curve (C) at steady state (s.s.), showing minor alterations; distal to (below) the curve (D) 5 min after bending, revealing a transient auxin dip. The local auxin maximum that forms after bending is found in the outer pericycle cell (B) at the bend, indicated with an asterisk (*). (E) Steady-state auxin concentration profiles of a root bent in the DZ showing an outer bias. (F) Steady-state auxin concentration profiles in the EZ, demonstrating the failure of the bend to cause a relevant increase in auxin. (G) Steady-state auxin concentration profiles in the MZ, showing the inversion of the inner/outer bias. Piece-wise linear color bar represents absolute and relative auxin concentrations; scale bar represents 100 μm.

When the model is extended to include the DZ, we observe distinct differences in auxin levels and flux patterns in the three regions of the root. In the MZ, auxin flowing down the vasculature towards the root tip moves back up the external cell layers, reentering the vascular flow via lateral transport across the width of the root. This reflux loop generates a strong accumulation of auxin around the QC. High auxin concentrations in this distal region drop exponentially in the proximal direction, towards the EZ (Figure 3A). The strong lateral PIN expression that allows auxin to flow from the external cell layers toward the vasculature results in very low auxin levels in the epidermis, cortex, and endodermis of the EZ. Consequently, in the vasculature, auxin concentration reaches a low, constant level, which is predominantly determined by the shoot-derived basal flux (red line in graph of Figure 3A). At the transition from the EZ to the DZ, lateral PIN1 and PIN2 expression decrease (Figure 2B and 2C). As a result, the basal flux through the vascular tissue, in which concentrations tend to be much higher than in the flanking external tissue files, becomes less confined (inset of Figure 3A and Protocol S1: Figure S2). Consequently, concentrations in the external cell files strongly increase in the DZ (green line in Figure 3A), and due to the upward flux through the external cell files and reflux back into the vasculature, an increase occurs in the vascular auxin levels (red line in Figure 3A). Although this result may seem counterintuitive (i.e., leakage of auxin from the vasculature into the external cell files causes an *increase* in the vasculature auxin concentrations), it can be readily understood by realizing that in equilibrium, net basal fluxes over different transverse cross-sections through the DZ, EZ, or proximal MZ should be equal. (The auxin decay rate is low, and therefore substantial amounts of auxin are lost only in the root tip, where concentrations are the highest, such that net basal fluxes in the DZ remain constant.) Consequently, any increase in the apical flow leads to an increase in the basal flow through the vasculature. A reflux is established, causing auxin on its way to the root tip to pass through the tissue multiple times, thereby increasing the concentration levels without changing the net downward flow (inset of Figure 3A and Protocol S1: Figure S2). At the transition from EZ to DZ, auxin levels in the exterior cells rise due to the weakened PIN expression. These higher levels are maintained throughout the rest of the DZ (Figure 3A). Along the whole straight root, concentrations are transversely symmetric. In summary, PIN proteins, whose expression is controlled by auxin concentration and by the root-tip–associated PLETHORA (PLT) transcription factors [23,24], produce different flux patterns in different root zones.

### Modeling Reveals That Curve Formation Is Sufficient to Bias Elevated DZ Auxin Distribution toward the Outside of the Curve

We investigated whether curve formation can trigger auxin accumulation by bending roots in silico (see Protocol S1: Text S1). When live roots curve, the cells on the inside of the curve are shorter than those on the outside, with the largest differences seen when roots grow in response to a gravitropic stimulus (Protocol S1: Table S4). Similarly, bending our model root alters the size and shape of cells in the curved region. Even when cellular PIN localization and the total amount of PIN activity per cell are held constant, the auxin distribution in the region of the bend rapidly changes. A new equilibrium situation is reached within 15 min (Figure 3B, 3C, and 3E). Auxin concentrations rise in both the vascular and external cell files, and a bias is established, with higher values in the outer half of the root and a maximum in the outer pericycle cells at the bend (Figure 3B, red line). This outcome is robust over wide ranges of diffusion and permeability values (Protocol S1: Figures S3 and S4), as well as for different cell-wall widths (Protocol S1: Figure S5) or mature cell sizes (unpublished data). Moreover, when PIN density per membrane length is held constant after a cell-shape change (rapid delivery of PINs to the membrane might provide such homeostatic control), bending the root still results in higher auxin concentrations in the pericycle on the outer half of the bend (Protocol S1: Figure S6). Simulations in which cell volume is held constant as the root is bent generate even stronger biases on the outer bend (Protocol S1: Figure S7). Furthermore, auxin maxima in the outer pericycle still form when the model is extended to include a Casparian strip, which might be auxin impermeable (Protocol S1: Figure S8), as lateral diffusion through the apical and basal cell walls has only a limited contribution to the overall lateral flux. Thus, our simulations indicate that curvature-induced auxin accumulation is a robust process that is not dependent on the particular choice of implementation, assumptions, or parameter values. Auxin levels rise due to the effect of cell-shape changes on auxin transport dynamics, with the extent of the increase depending on the degree of curvature (Protocol S1: Figure S9). The localized increase in auxin concentration is independent of the mechanism that induces the curve, such as the gravitropic response.

Given that root bending results in cell-shape changes, it is tempting to think that auxin accumulation is the result of cell-length differences only. However, that is not a sufficient explanation. For a cell with polar PIN expression (i.e., an epidermal, cortical, or vascular cell), the mean auxin concentration is expected to increase linearly with cell length. This is strongly dependent on the fact that cells are discrete units in which the flux becomes “boosted” at each cell–cell transition due to the unidirectional auxin transport. Thus, within the cell, auxin forms a gradient along which, due to passive diffusion, auxin fluxes polarly. (For an ideal one-dimensional vascular cell: given the cytosolic diffusion constant of auxin *D*, the effective polar transport *P* across the cell, and an auxin concentration *a* at the basal end, a linear gradient will establish within the cell with a slope *Pa/D*. Consequently, given the same auxin transport properties, the slope does not depend on the cell length, whereas the mean auxin level within the cell—given by *a*(1+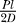 ), where *l* is the length of the cell—increases with length. Similarly, under the assumption of constant basal influx *i*, the mean auxin level within the cell is given by *i*(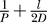 ). Thus, length increase (in both cases) is expected to result in a linear rise in mean auxin concentration. Although it cannot be excluded that such small auxin increases could suffice to trigger lateral root initiation, this mechanism by itself is insufficient to explain all of our observations. First, geometric considerations alone would imply that the cells along the inner side of a bend, which have become smaller due to the bending, should present lower concentrations. Clearly, this is not the case: Figure 3B and 3E shows an increase in auxin in all cell files at the bend, in agreement with our experimental observations (Figure 1G, black lines). Moreover, holding the degree of curvature constant over a bend, but varying the length of the region undergoing bending, progressively increases the bias in auxin concentrations (Protocol S1: Figure S10), indicating that tissue properties are also important in generating the bias. Simulations of J-hooks that cause only 6% differences in cortical cell lengths still present remarkable increases in auxin levels and bias, but only when the length of the bent tissue is sufficiently long (Protocol S1: Figure S11).

The formation of a strong bias results from an increase in the amount of auxin “leaking” out of the vasculature into the external cell files. Given the large fluxes that pass through the vascular tissue, internal auxin gradients are steep within the vascular cells. Steeper gradients imply larger auxin increases due to cell-length increase, and as a result, the concentration difference between vascular and external cell files is enhanced due to bending, leading to the lateral flow. As is the case for the DZ in straight roots (insets of Figure 3A and Protocol S1: Figure S2), this higher concentration of auxin in the external files locally enhances the DZ-reflux loop, causing auxin levels to further rise in the vasculature. This is accompanied by a further increase in lateral flux, effectively recapturing even larger amounts of auxin that would otherwise pass through the region only once (Protocol S1: Figure S2). The result is an increase in auxin concentration at the bend. To untangle the relative contributions of cell shape and auxin reflux, we analyzed two situations in which transport in the bent region is locally interrupted. First, we introduced an impermeable vertical wall in the apoplastic space in between the endodermis and pericycle to the model in the region of the bend (Protocol S1: Figure S12A and S12C). This prevents lateral fluxes between vascular and external cell files at the bend, and as a result, the increase in auxin is reduced in the vasculature and outer external cell files, as well as lacking in the inner external cell files. Second, we completely eliminated auxin reflux by blocking fluxes through the external cell files (Protocol S1: Figure S12B and S12C). This leads to an even stronger reduction in auxin at the bend, revealing the importance of the reflux loop in this mechanism.

To determine how robust the tendency for lateral root formation to occur on the outside of a curve is, we examined the position of emerged lateral roots in mutants with altered growth patterns. The *wag1/wag2* double mutants have a wavy root phenotype, and *rcn1–6* plants exhibit a pronounced skew, with root tips slanted to the right when plates are viewed from the front. Both mutants maintain a strong outward bias in the location of lateral root formation. All of the roots bearing emerged laterals on the curve 3 d after inversion had those laterals on the outside of the curve (44 out:0 in for *wag1/wag2* and 46 out:0 in for Col-0 in one experiment; 33 out:0 in for both *rcn1–6* and Col-0 in a second experiment).

Because flux patterns differ substantially throughout the root tissue (insets in Figure 3A), the different zones present specific responses towards bending (Figure 3E–3G). These differences follow naturally from the differences in PIN abundance: The MZ, due to its large lateral fluxes and steep auxin gradient, not only fails to generate the typical bias observed in the DZ, but actually forms an inverted bias (higher levels on the inside, i.e., concave side), without the appearance of a single maximum (Figure 3G). The EZ is capable of generating a small outer/inner bias (Figure 3F), due to the effect of increased cell lengths (similar to Protocol S1: Figure S12B), but the reflux that is essential for creating a substantial increase is suppressed by strong lateral PIN expression. These lateral PINs act like a “cordon” along the vasculature, keeping auxin levels within the external cell files very low. Thus, PIN organization in the different zones (MZ, EZ, and DZ) explains why lateral root formation is limited to the DZ.

Classical physiological experiments have shown that tip removal induces lateral root formation, even when the shoot is decapitated, as long as either a sufficiently long root segment is preserved or external auxin is supplied [25]. We simulated the same treatment in our model, and observed auxin accumulation dynamics that corroborate the link between vascular auxin accumulation and lateral root initiation (Protocol S1: Figure S13).

### AUX1 Generates a Positive Feedback Loop Leading to Lateral Root Initiation

**Figure 4:**
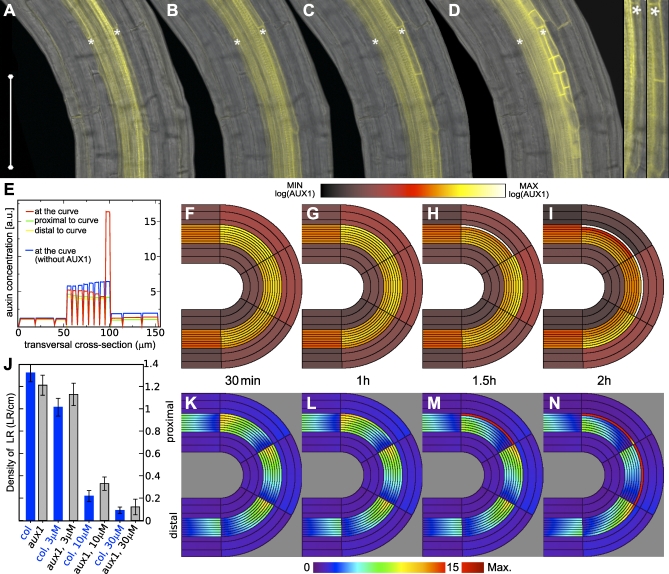
AUX1 Affects Lateral Root Initiation (A–D) AUX1-YFP accumulates uniformly in the pericycle cells on the outside of the curve prior to lateral root initiation. (A) 230 min before, (B) 90 min before, (C) 10 min after, and (D) 520 min after the first asymmetric cell division. Insets from (A) and (C) show AUX1 levels in a single founder cell. Left shows a blowup of (A); right, a blowup of (C). White asterisks indicate the pericycle cell files. (E) Simulation showing the effect of a 4-fold increased influx in the two most apical pericycle cells of the outer bend, resulting in a local maximum, shown by comparing the transversal profile through an AUX1-expressing cell row (red) with cell rows proximal (green) and distal (yellow) to the bend. Default bias caused solely by curvature is shown in blue. (F–I and K–N) Simulation in which the whole tissue is endowed with the same sigmoidal auxin-dependent AUX1 response; (F–I) show the increase in magnitude of the AUX1 response after bending that eventually becomes focused to the outer pericycle cells, using a logarithmic color map from black (no AUX1 expression) to white (high AUX1 expression), as indicated in color bar; (K–N) show the resulting corresponding auxin concentration profiles, presenting a localization and amplification of the maximum. Heatmap for auxin concentrations indicated below; 30 min (F and K), 1 h (G and L), 1.5 h (H and M), and 2 h (I and N) after root bend. (J) 1-NOA inhibits lateral root formation, with wild-type plants being more sensitive than *aux1* mutants. Density of emerged lateral roots was determined 4 d after roots were transferred to fresh media, for that region of the root that grew after transfer. Error bars represent the standard error of the mean (SEM).

**Figure 5:**
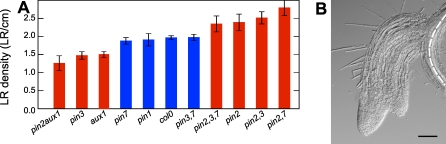
PIN Proteins Affect Lateral Root Density and Spacing (A) Density of emerged lateral roots (LR) was measured for plants grown on vertically oriented agar plates, 7 dpg. Red color indicates a statistically significant difference from Col-0 (*p* < 0.05 in a Student *t*-test). Error bars indicate SEM. (B) *pin2,3,7* root showing fused lateral roots; white dotted line indicates the main root axis. Scale bar represents 100 μm.

Seedlings with loss-of-function mutations in AUX1 have decreased numbers of lateral roots [10] (Figure 5A), resulting from reduced rates of lateral root initiation [12]. To investigate how changes in AUX1 might be associated with lateral root formation, AUX1:YFP plants were gravistimulated for 4 h and then subjected to dynamic imaging. AUX1:YFP levels accumulated asymmetrically in the region of the bend, with levels on the outside of the curve being clearly higher than those on the inside (Figure 4A–4D; Video S2). Fluorescence intensity within the pericycle increased steadily before and after the first asymmetric cell divisions. Notably, the distribution of AUX1:YFP was relatively uniform within a single pericycle cell membrane (Figure 4C). The first cell divisions took place 2–3.5 h after imaging began, thus the increase in AUX1 along the vasculature occurs no later than the increase in auxin response in the founder cells, and likely before it (Figure 4A–4D).

We noted striking similarities between AUX1 and auxin response reporter accumulation. Transcription of AUX1 is substantially up-regulated in roots within 90 min after auxin application [26]. Auxin application to AUX1:YFP plants results in increased levels of fluorescence in the pericycle and ectopic expression in cells outside the vasculature. These changes become visible within 2 h after auxin application, and membrane localization of AUX1 is pronounced within 3 h (Protocol S1: Figure S14, compare S14B, S14C, and S14F), indicating that auxin induces AUX1. Because AUX1 facilitates auxin influx, this response not only provides a mechanism for increasing the auxin concentration within the responding pericycle cells, but also initiates a positive feedback loop in which those increased concentrations go on to further induce AUX1.

A major role for vascular AUX1 in lateral root formation seems at odds with previously published data, which revealed that AUX1 expressed under the control of the J0951 GAL4 enhancer trap line rescues the lateral root phenotype [5,18]. Near the root tip, this line drives expression in the expanding epidermis and root cap, as was reported. However, we found that in mature portions of the root, J0951 mainly drives expression in the vasculature (Protocol S1: Figure S15), consistent with the most parsimonious hypothesis from the expression data that this is the primary site of action for lateral root induction. Another concern about postulating a major role of auxin influx in lateral root initiation is that the *aux1* mutation has only a mild effect on lateral root initiation. To assess potential redundancy, we administered 1-napthoxyacetic acid (1-NOA), an auxin analog that lacks auxin activity and inhibits AUX1-mediated transport. 1-NOA inhibits AUX1 and LAX3-mediated transport [27,28], but polar transport of ^3^H-IAA in inflorescence stems remains unaffected, indicating that it does not affect PIN efflux proteins [29]. 1-NOA inhibits lateral root formation when assayed 4 d after transfer; the density of emerged lateral roots on the new growth declined steadily with increasing concentrations of 1-NOA (Figure 4J). Wild-type plants showed a statistically significant reduction in lateral root formation after treatment with 3 μM 1-NOA, whereas *aux1* roots do not. Thus, 1-NOA acts on AUX1. Higher concentrations of 1-NOA do generate a significant reduction in lateral root density in *aux1* plants, confirming that 1-NOA also acts on other members of the AUX/LAX family of auxin import facilitators. This suggests that such members are active in lateral root initiation, although we cannot exclude the possibility that emergence is affected, as recent evidence indicates that LAX3 promotes lateral root emergence [28]. Whereas 96% (23/24) of wild-type roots that were manually curved formed lateral roots on regular media, only 50% (12/24) of roots did so when placed on 30 μM 1-NOA. Roots that are transferred to 1-NOA and left straight only develop emerged lateral roots 32% of the time, indicating that curve formation is still a positive stimulus for lateral root formation in the presence of the 1-NOA. For roots that did form, 11/12 were on the outside of the curve, indicating that bias toward the outside was still maintained.

We next investigated a positive feedback role for AUX1/LAX proteins in auxin accumulation, using the modeling approach. Simulations in which AUX1 is expressed at high levels in specific pericycle cells on the bend result in a clear increase in auxin in these cells (Figure 4E). Motivated by this result, we investigated whether high expression of AUX1 in outer pericycle cells could emerge as a consequence of the flux patterns, without invoking special rules for the pericycle. We built a simple feedback into the model, in which AUX1 expression in the DZ cells is regulated by a sigmoidal response to auxin concentration (see Protocol S1: Text S1 for details). A self-organizing amplification of auxin accumulation and AUX1 expression occurs through their mutual feedback. Consistent with our experimental observations (Figure 4A–4D), these simulations reveal increased AUX1 concentrations only upon curvature (Figure 4F–4I and 4K–4N, and compare Video S3 with Video S4, for control). The resulting auxin maximum becomes localized at one to three outer “pericycle” cells because, as a result of these cells' strategic position at the interface of the basal-directed vascular flux with the flanking external apical fluxes, the gain of auxin due to a certain increment in AUX1 expression levels is greatest in these regions. As cells “compete” for auxin uptake through AUX1 and its auxin-dependent regulation, a winner-take-all situation manifests, with pericycle cells having a clear advantage. The AUX1 response may spread in a cell-wise fashion to distal neighbors (Figure 4H and 4I) within the bent region. When AUX1 expression levels become very high in a pericycle cell, this cell begins to efficiently take up auxin from the cell wall, thus depleting neighbor auxin concentrations. Subsequently, auxin concentrations within the cell accumulate and its basal efflux (due to the basal PIN expression) becomes sufficient to replenish the neighboring distal pericycle cell. The neighbor, through its AUX1–auxin regulation loop, can initiate a similar cycle, also reaching high auxin levels. This effect tends to be restricted to the bend, where the auxin maxima become fixed. In some simulations, we also observe an auxin maximum that travels down the root towards the meristematic region in the form of a pulse, initiated from the newly formed curvature (Video S5). This suggests that the formation of a curve could potentially trigger periodic increases in auxin concentration in regions as far away as the basal meristem region. Importantly, simulation of the AUX1 feedback cannot increase “pericycle” auxin levels in straight roots (Video S4). Collectively, our data reveal that modest increases in auxin concentration on the outside of a curve are amplified by the AUX1/LAX auxin influx facilitators, serving as a central mechanism for lateral root initiation. An initial bias, supplied here by the bending of the root, is necessary for the positive feedback. The mechanism, however, readily accepts other sources of bias, predicting in all cases auxin accumulation in the pericycle cells.

### Auxin Transport Mutants Impact Lateral Root Density in Nonadditive Ways

The density of emerged lateral roots depends on the presence of specific auxin transporters [30]. We noted that slight changes in growth circumstances have large effects on lateral root densities, so in the following analysis, we compared the density of lateral roots in wild-type and mutant plants that were grown on the same plates. At 7 d postgermination (dpg), statistically significant increases in lateral root density were seen in *pin2* plants (Figure 5A), whereas *pin3* mutants showed a significant reduction. *pin7* and *pin1* loss-of-function mutations did not appreciably alter lateral root density. Although several mutations affect the frequency of lateral root formation, they do not interfere with the tendency to form a lateral root on the outside of a curve (Table 1). The effect of AUX1 on lateral root density decreases with age [3], an observation that is substantiated by our data (Protocol S1: Figure S16), despite the fact that data in Figure 5A and Protocol S1: Figure S16 were obtained in different laboratories. Such age-related differences might be caused by compensatory mechanisms and/or changes in root geometry that occur as a result of continued growth (see Protocol S1: Figures S10 and S11 for discussion). *aux1 pin2* double mutants mimic the effect seen in *aux1* (Figure 5A), indicating that the loss of AUX1 has a stronger influence on lateral root formation than does PIN2, supporting our conclusion that AUX1-mediated auxin influx is central to lateral root positioning. Double mutants reveal unexpected reversals in density changes (Figure 5A), suggesting nonadditive interactions between auxin transporters in lateral root formation.

**Table 1:**
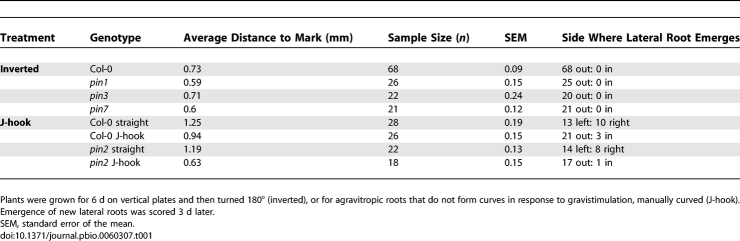
The Bias for Forming Lateral Roots on the Outside of Curves Is Maintained in PIN Mutants

To investigate the apparently complex influence that PIN proteins have on lateral root density, we took advantage of the possibility of the model to independently alter lateral, apical, and basal expression of PIN proteins (Protocol S1: Figure S17A and S17B). The potential for forming lateral roots upon curvature is increased slightly when the lateral PIN expression component is diminished (i.e., “lateral” mutant), and increasingly so for a “basal” mutant, both due to a more effective auxin reflux. In contrast, an “apical” mutant has a somewhat decreased potential for auxin accumulation at the bend, because reduced upward flow in the external cell layers reduces the reflux. Interestingly, when combining these in different permutations, forming double and triple “mutants,” nonadditive effects occur (Protocol S1: Figure S17C and S17D). This is a consequence of the complex manner in which PIN expression modulates reflux loops within the root. We find the strongest propensity for lateral root formation in the “basal-lateral” mutant; a prediction that cannot be readily tested, as PIN2 is expressed both apically and laterally, and it is as yet impossible to uncouple these two components. Despite this difficulty in experimental validation, our simulations corroborate the observation that changes in PIN transporters play a significant and nonlinear role in the lateral spacing of root primordia.

### Lateral Inhibition Is Modulated by PIN Proteins

Longitudinal spacing of lateral roots is diminished in *pin2,3,7* triple mutants. These mutants have marked increases in lateral root density not correlated with root length (Figure 5A). In 13/100 of these roots, we observed lateral roots that formed exactly adjacent to one another or that fused at the base, separating into two distinct roots only near their tips, a phenotype that was never seen (0/65) in wild-type roots. The existence of adjacent and fused laterals in *pin2,3,7* triple mutants indicates that mechanisms leading to lateral inhibition of organ formation are interrupted in those plants (Figure 5B). Consistent with this finding, modeling simultaneously decreased vascular flow and reduced flow through the epidermis (lateral-apical mutant) largely mimics loss of PIN2,3,7 and leads to a higher accumulation of auxin at the EZ–DZ boundary as well as higher accumulations due to bending in the region of the curvature, thus predisposing a larger region for lateral root induction (Protocol S1: Figure S17D–S17F).

**Figure 6:**
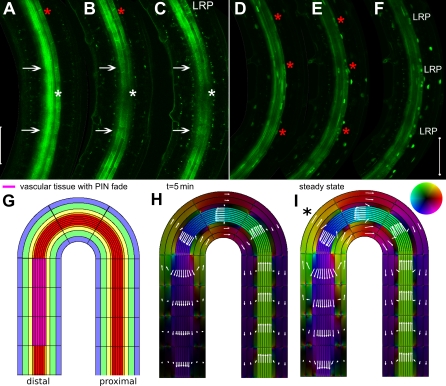
PIN3/7 Modulate Longitudinal Positioning of Lateral Roots (A–C) Depletion of PIN7:GFP in the stele is followed by primordium (LRP) initiation on the apical end of the depletion zone. Arrows indicate central portion of this zone. White asterisk indicates outer pericycle cell file. (D–F) Uniform PIN7:GFP expression correlates with initiation of multiple primordia around the bend. Red asterisk, placed just external to the pericycle cell file, indicates a cell that will undergo division. Nuclear localized DR5:vYFP indicates regions of auxin response. (A and D) 300 min, (B and E) 100 min, and (C and F) 0 min prior to cell division. (G) Segment of bent region in model; vascular cells with depolarized and weakened PIN expression are shown in pink. (H and I) Dynamics of flux field as a result of bending and fading PINs in distal cells. Flux directions are represented through angle dependent colors as indicated in the color-circle; flux magnitude is set through the color intensity, using a log-scale (from black, no flux, to bright color, maximum fluxes), i.e., the radial component of the color-circle.

In the mature region of a straight-grown root, PIN3 and PIN7:GFP are similarly expressed in the vasculature. In cases in which single lateral roots form, the levels of PIN3 or PIN7 fluorescence decrease, and the signal becomes diffuse throughout the cytoplasm. This decrease in polar localization occurs in a region that extends across the width of vasculature, followed by the formation of a single lateral root primordium just on the shoot side of that region (Figure 6A–6C, Video S6). PIN3 and PIN7 are transcriptionally regulated by auxin [31], and by the tip-focused PLT gradient [24], and severing the main root from the shoot, which serves as its primary source of vascular auxin, decreases the level of PIN7:GFP within about 5 h. Interestingly, when the distribution of PIN3 or PIN7 did not decline, multiple primordia formed along the curve. Typically, all pericycle cells located on the outside of such a curve underwent an initial round of cell division (Figure 6D–6F) . Thus, PIN3:GFP and PIN7:GFP during lateral root initiation document a strong correlation between PIN reduction and the efficiency of lateral inhibition.

Our model predicts that simply bending a root in the DZ causes auxin concentrations to drop just distal to the curve, especially in the vascular tissue (Figure 3D, yellow line). This strongly suggests that bending might be the source of the decreased auxin levels distal to the curve, leading to partial depolarization of PIN3 and PIN7, although the molecular mechanism regulating PIN modulation remains uncharacterized. In simulations with decreased basal expression and partial depolarization of vascular PINs, basal vascular fluxes are interrupted (Figure 6G) and directed towards the external cell files (Figure 6H and 6I). As a result, more auxin is brought into the apical stream, in the direction of the shoot and the bend. Auxin reenters the vasculature at the bend, where basal PIN expression is unaffected, leading to a greater accumulation of auxin in the curved region. Importantly, this process can regulate the spacing between auxin maxima. This is consistent with our experimental observations that lateral inhibition is suppressed in the absence of this PIN3/7 fading, suggesting that the feedback between PIN and auxin can explain the suppression of nearby emerged lateral root initials. Through AUX1 feedbacks alone, these distances would not be maintained. On the contrary, multiple cells along a curve could be expected to differentiate, due to comparably high auxin values.

The model further predicts that the location of the auxin maximum relative to the center of a curve depends on the relative contributions of AUX1 and PIN3/7. If the AUX1 response occurs more rapidly than the decrease in PIN3/7, the auxin maximum is focused on the proximal region of the bend, but is able to spread longitudinally along the outer pericycle cells at the curve (Video S3). Alternatively, if the decrease in polarly localized PIN3/7 precedes the AUX1 increase, it focuses the maximum to the distal region of the bend. This is in full agreement with the observation that decreases in polar PIN3/7 are highly correlated with formation of one lateral root just proximal to the fading.

## Discussion

Here, we show that curvature is causal for lateral root initiation, an observation that extends the previously observed correlations between curves and sites of lateral root formation [4,6,18]. In vivo observations indicate that auxin accumulates on the outside of the vasculature in bent regions of the root, the same area in which lateral root initiation is favored. The auxin influx carrier AUX1 is increasingly up-regulated in this region, and becomes focused in either one or several cells in tight correlation with the extent of down-regulation of two PIN efflux facilitators expressed in the vasculature. Mutation of AUX1 and pharmacological inhibition of the AUX1/LAX pathway confirm a major role for the auxin influx pathway in determining the density of root primordia.

Our novel multilevel feedforward model for lateral root initiation parsimoniously explains both the patterning mechanism that restricts a new auxin maximum to a local site and the zone of competence for lateral root formation. We show that the presence of a curve leads to localized cell-shape changes, which produce a “trigger” for the auxin transport system in the DZ, which acts as an excitable system. Activation of the trigger results in increased levels of auxin response on the outside of the vasculature, exactly at the “pericycle” boundary layer where lateral roots form. Once small changes in auxin concentration occur, they can change the expression and polar localization of auxin transporters [32–34]. We observe an AUX1 positive feedback loop and down-regulation of PIN3 and PIN7, both of which can be seen as self-organizing, cell autonomous processes, triggered by an initial bias in auxin concentration, that further amplify the trigger. Employing both experimental work and predictive modeling has proven powerful, leading to results that would not have been obtained by either method alone. Auxin fluxes are not simply a “readout” of the PIN localization, but depend on local intracellular and cell-wall auxin concentrations, as well as on global tissue structure. In addition, positive feedback and cross-regulation between auxin accumulation and AUX1/PIN transporter activity play an important role in lateral root patterning.

Our model explains how a specific trigger, i.e., curvature, can provoke developmental responses in a self-organizing fashion. Our model involves local cues that are causally linked to lateral root formation, and in this respect, differs from previous suggestions for a role for the MZ/EZ boundary region based on correlations between auxin response oscillations in this region [5]. Our study and the work of Lucas et al. [18] indicate that fixed-period oscillations are not required for lateral root formation. Our simulations show traveling pulses of auxin to the root tip associated with primordia formation, suggesting that oscillations in auxin within the vasculature at the MZ-EZ boundary, as observed by De Smet et al. [5], might be a consequence of formation of previous lateral root primordia rather than a cause for the formation of new ones. The mechanism that we propose has two modules: the initial cell-size trigger and the excitable DZ-specific AUX1/PIN feedback loop. It is important to note that several additional triggers may act on the feedback loop to stimulate lateral root formation. For example, mechanosensory triggers or nutrient-induced changes in auxin transport and response may impinge on the activity of the DZ loop. Future research should address whether such additional inputs are needed to explain the root branching pattern in its complex natural environment.

The model for explaining rhizotactic patterning that we present here is similar to current models for phyllotactic patterning [21,35–38] in the sense that organ formation is controlled by the abundance and location of auxin transporters that collectively generate an auxin maximum. The important role for AUX/LAX proteins in rhizotaxis is mirrored by the recent discovery that these proteins are redundantly required for phyllotaxis [38]. Whereas the initial events and the feedback loops leading to auxin accumulation maxima in the shoot are incompletely understood, there is one more intriguing parallel: it has been observed that leaf formation that follows from local auxin accumulation can be initiated by local increases in cell expansion [39,40]. Thus, a biophysical mechanism that alters relative cell shapes in a tissue, changing local flux patterns and followed by responsive changes in auxin transporters, may be common to both root and shoot organ formation. This suggests that a unified concept for organ initiation in root and shoot systems may be within reach.

## Materials and Methods

### Plant materials and phenotypic analysis.

Arabidopsis thaliana plants, ecotype Col-0, were used for all experiments, including marker lines and auxin transport mutants. The DR5vYFP marker line was generated by fusing DR5rev [41] to nuclear targeted venusYFP coding sequence [15] in a pGREENII vector. Transgenic plants were generated by transforming Col-0 wild type as described [42]. PIN1:GFP, PIN2:GFP, PIN3:GFP, PIN7:GFP, and AUX1:YFP marker lines and auxin transport mutants were as described [43]. Unless otherwise indicated, plants were grown on vertically positioned GM plates containing 1% sucrose and 1.5% agar.

For reversion experiments, marks were placed on the agar beside the root tips of 6-d-old plants. Plates were turned 180° and left for various periods, after which they were returned and left for 3 d. Distance from the center of the newly formed curve to the nearest emerged lateral root and degree of root curvature were measured using ImageJ (v. 1.36 and 1.38); for more details, see Protocol S1: Figure S1. If no lateral roots emerged within the image frame, the distance to the nearest lateral root was set to the longest recorded length. For manual root curving experiments (J-hooks), 6-d-old plants were grasped with tweezers just below the cotyledons and turned in a J shape by gently dragging the root across the agar surface with the center of the curve 0.5 cm from the root tip. Control roots were also grasped and moved along the agar, but were left in a straight orientation. Marks were made on the agar beside each root, 0.5 cm from its tip. For lateral root density measurements, the number of emerged lateral roots was counted with the aid of a dissecting microscope, and root lengths were measured as described above for plants 7 dpg and 12 dpg. Germination was monitored every 12 h. We noted that lateral root density is very sensitive to changes in environmental conditions, and we controlled for this as closely as possible within each experiment. For example, wild-type and mutant plants were grown on different sides of the same plate. Data for Figure 5A and Protocol S1: Figure S16 were obtained from plants grown in different laboratories. 1-NOA treatments were performed by transferring 6-d-old plants to fresh agar plates with the indicated concentrations; addition of 30 μM 1-NOA was as described [44], resulting in 0.006% DMSO in the media; control plates contained comparable amounts of DMSO. Lateral root densities were determined for the newly grown portion of the root: the entire MZ and EZ, together with the portion of the DZ formed during the interval indicated, and hence, the densities reported in Figure 5A are not comparable to those of Protocol S1: Figure S16 in which the whole length of the root was considered.

### Cell-length measurements.

Cortical cell lengths were obtained from plants that were either inverted 180° and allowed to grow their own curve, or hand curved into the shape of a J. Roots were imaged under a Zeiss confocal microscope fitted with a 25× objective. To assist in holding the curves in place, coverslips were coated with a thin layer of agar in MES prior to use. During imaging, a slab of agar was used in place of a slide or coverglass. Projection errors and root twisting make it difficult to obtain accurate measurements of cell length. To minimize that source of error, and provide a better estimate of actual cell length, cortical cells were measured only if their end walls were in focus when the xylem plane was clearly visible. Lengths were obtained from images using Zeiss software. The radius of curvature was determined by fitting a circle to three points: the center of the measured area and the two most distant end walls of the specific outer cortical cells that were measured. If only one cortical cell was measured, the region of the cortex under consideration was extended by 200 μm to minimize error in curve fitting.

### Microscopy.

Confocal microscopy was performed using an inverted Zeiss confocal microscope, and image analysis was done using Zeiss LSM Pascal (3.2SP2) software. For live imaging, 6-d-old plants were transferred to coverslip-bottomed imaging chambers containing water or various IAA dilutions, and covered with a slab of agar. Prior to transfer, some roots were either manually curved or gravitropically stimulated by inverting the plates for 4 h. Images were taken every 10 min for a period of 14–16 h, with an autofocus system employed.

### Mathematical/Computational modeling.

*Auxin dynamics.* Simulations are performed by numerically solving transport equations through an implicit reaction-diffusion algorithm. It takes into account diffusion within cells and cell wall at a rate of 300 μm^2^/s, as well as permeability across a cell membrane: influx permeability is by default set to 20 μm/s, but is 4-fold increased when AUX1 overexpression is taken into account; efflux permeability is set to 1 μm/s in the absence of visible PINs, and to 5 μm/s or 20 μm/s in the case of weak, respectively strong PIN expression. Boundary conditions at the proximal end of the root segment are kept such as to mimic the continuation of the described segment, being linked to the rest of the plant. Further details on the implementation of auxin transport can be found in Grieneisen et al. [22], and in Protocol S1: Tables S1–S3 and Text S1.

*Root bending.* Root bending has been implemented through a geometrical transformation in which curvature, axis of rotation, and length of curved segment can be specified and controlled. Bending is always applied in such a way that the length along the central axis of the root does not change. Consequently, due to the bending, cells on the inner side of the curve decrease in length, while those on the outer side expand. All cells keep their original width. To ensure that cell-length changes do not affect the total amount of PIN molecules along a cell membrane, lateral PIN-dependent efflux is diminished proportionally to the gain of length due to bending. (Note that using an alternative assumption, in which PIN density remains constant independent of the addition of the cells perimeter, will continue to yield similar results, as we show in Protocol S1: Figure S6). Graphs showing transversal and longitudinal auxin profiles of bent roots are obtained by first defining for an unbent root the horizontal lines that pass through the centers of the cell rows and the vertical lines through the centers of the cell files. During bending, these lines then undergo the same transformation as the root itself, thereby creating a link between the positions after bending and the corresponding locations along the original lines in the unbent case. In all plots that present auxin concentration profiles along cell files or cell rows, this mapping of the positions from the original (unbent) to the bent state is being used. This explains why the cell lengths within the inner and outer regions always appear to be the same when plotted on graphs. Although this method slightly modifies the length along bent cell files on the graph plot, it is preferred because it makes direct comparison between cells before and after bending possible.

*AUX1 regulation.* The feedforward regulation of AUX1 by auxin is implemented by using a moving average of the intracellular auxin concentration. The variable *C_i_(t)* gives the mean auxin concentration within cell *i*, integrated over a time window Δ*T_AUX_*_1_ (typically 10 min). It determines the level of AUX1 expression, *A_i_(t)*, of that cell *i* at a given moment *t*, through the sigmoidal relation


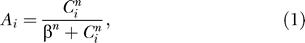


where β is the auxin concentration at which AUX1 expression reaches half its maximum response, and *n* determines the steepness of the response. The AUX1 expression enhances auxin influx along all sides of the cell. To demonstrate that changes in auxin alone are sufficient to regulate AUX1 expression, we use the same functional response and the same parameters for all cells of all cell types within the DZ, both within and outside of the bent region. In short, the expression of the AUX1 auxin importer dynamically changes as a consequence of the absolute levels of auxin within the same cell. (Note that only local information is being used, i.e., for example relative differences in auxin levels between neighboring cells do not play a role.) In turn, (changes in) auxin concentrations result from (modifications of) local fluxes, which are influenced by the influx rates along the membrane of the cell and its close neighbors. At each position along the membrane of a cell *i*, the influx is given by


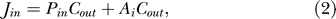


where *P_in_* is the default influx permeability, *A_i_* the permeability due to AUX1 expression, and *C_out_* the local concentration within the cell wall, just outside the membrane. This feedforward loop, however, is not inherently unstable (see Video S4). This is because AUX1 up-regulation reduces the reflux, and thereby the total auxin throughput.

## Supporting Information

Protocol S1 Details of Modeling, and Supplementary Figures and Tables (9 MB PDF)

Video S1 Increased Auxin Response Precedes Asymmetric Division of the Founder Pericycle Cells DR5:vYFP root was gravitropically stimulated for 4 h, then placed horizontally and imaged every 10 min thereafter. Arrow indicates approximate height at which an endodermal cell shows increased auxin response prior to the strong increase in founder cells. The root is oriented such that the top of the frame is closest to the shoot, bottom to the root tip.
(2.02 MB MPG)

Video S2 AUX1:YFP Levels Increase in Pericycle Cells on the Outside of a Curve prior to Asymmetric Cell Division The root was gravitropically stimulated for 4 h prior to imaging; frames taken 10 min apart. The root is oriented such that the top of the frame is closest to the shoot, bottom to the root tip.
(2.02 MB MPG)

Video S3 Simulation of AUX1 and Auxin Feedback, Showing Regulatory Dynamics and Auxin Changes after Root Bending Left panel: AUX1 expression levels indicated by color bar above. Right panel: auxin concentration levels, indicated through heatmap color bar above. Scale bar represents 100 μm. Time after root bending is indicated.
(5.29 MB MPG)

Video S4 Control Simulation of AUX1 and Auxin Feedback within a Straight Root, Confirming Stable Regulatory Dynamics in the Absence of Curvature Upper panel: AUX1 expression levels indicated by left color bar. Lower panel: auxin concentrations indicated by heatmap on the right. Scale bar represents 100 μm. Simulation is started with a root devoid of auxin. Time after root bending is indicated.
(2.12 MB MPG)

Video S5 Simulation of AUX1 and Auxin Feedback, Showing Regulatory Dynamics and Auxin Changes after Root Bending over the Whole Extension of the Root Downstream effects due to root curvature can be observed that travel through the root in the distal direction. Left panel: AUX1 expression levels indicated by color bar above. Right panel: auxin concentration levels, indicated through heatmap color bar above. Scale bar represents 100 μm. Time after root bending is indicated.
(5.29 MB MPG)

Video S6 PIN7:GFP Marker Line Showing a Decrease in Fluorescence in a Region of the Vasculature Preceding the Formation of a Single Lateral Root Primordium Lateral root primordium forms on the shoot side of the region with decreased fluorescence (indicated by arrow); the root is oriented such that the top of the frame is closest to the root tip. The root was gravitropically stimulated for 4 h prior to imaging; frames were taken 10 min apart. Punctate fluorescence that accumulates over time, here seen especially in the cytoplasm of cortical and endodermal cells, was also observed when untransformed roots were imaged this way, and thus is not part of the GFP signal.
(2.30 MB MPG)

## Abbreviations

dpg: days postgermination
DZ: differentiation zone
EZ: elongation zone
MZ: meristematic zone
1-NOA: 1-napthoxyacetic acid
PLT: PLETHORA
