# Correction: Root System Architecture from Coupling Cell Shape to Auxin Transport

**DOI:** 10.1371/journal.pbio.1001984

**Published:** 2014-10-10

**Authors:** The PLOS Biology Staff

Since publication of this paper, the authors became aware of details in the preparation of a composite figure that required correction.

In the previous version of Figure 2, a segment within a cortical cell close to the shootward end of panel 2D1 was processed to match between overlapping images, which does not fully comply with image processing standards. We have reconstructed all composite panels in the corrected Figure 2 from the raw data to ensure that the figure is now fully compliant. In this process, we have slightly shifted the positioning of some inset boxes for an improved positional match with the high magnification images they are referring to. None of these changes affect the results and conclusions reported in the paper in any way. The correction version has been provided here.

**Figure 2:**
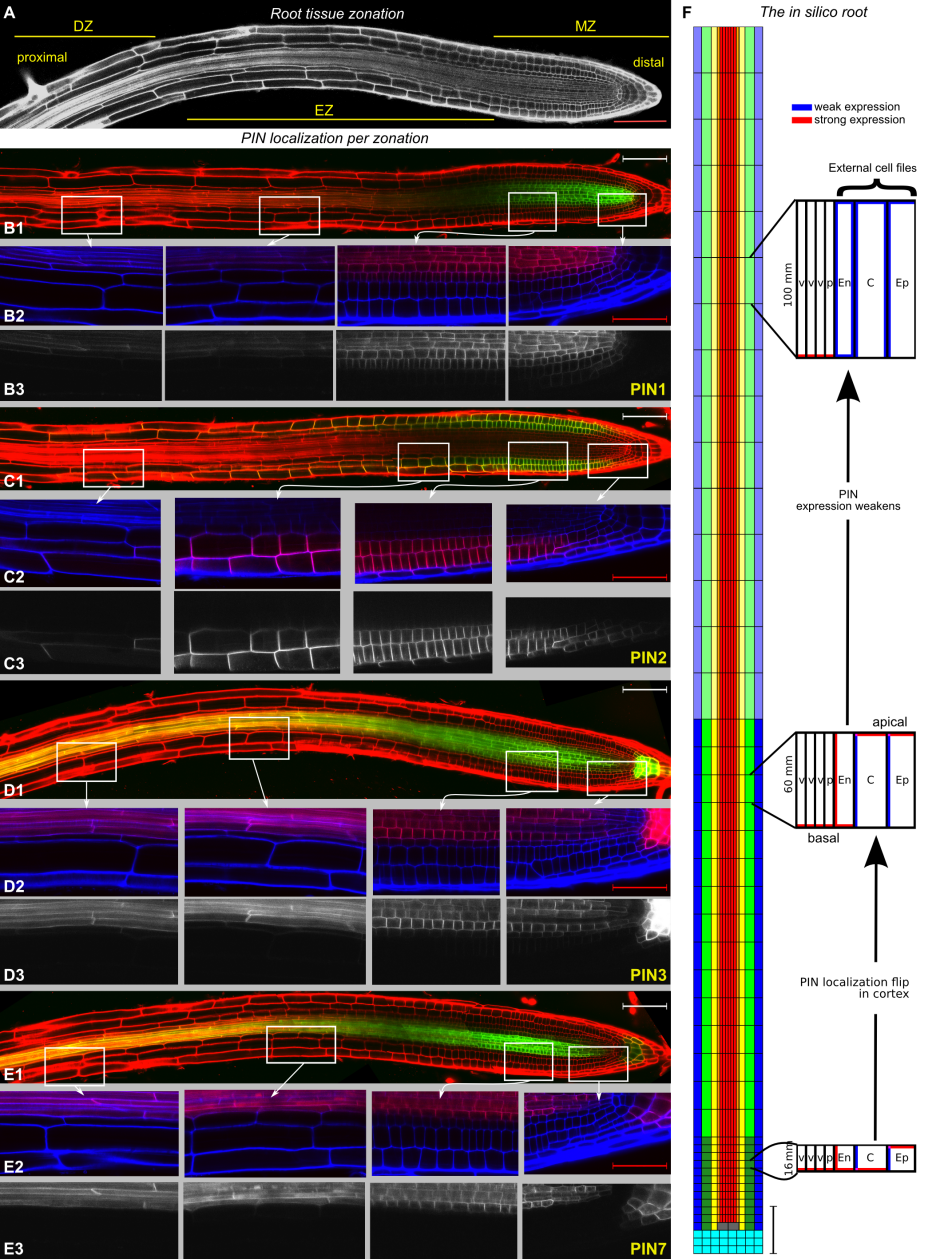
Root and Model Layout. (A) Image of a live root, with meristem (MZ), elongation (EZ), and differentiation (DZ) zones indicated.(B–E) PIN expression domains of (B1–B3) PIN1:GFP, (C1–C3) PIN2:GFP, (D1–D3) PIN3:GFP, and (E1–E3) PIN7:GFP. For B1, C1, D1, and E1, the GFP is shown in green and the propidium iodide (PI) stain in red. In B2, C2, D2, and E2 (enlargements of the insets of the DZ, EZ, and MZ in overviews B1, C1, D1, and E1, respectively), the GFP is shown in red and PI channel in blue. In B3, C3, D3, and E3 (enlargements of the insets of the DZ, EZ, and MZ in overviews B1, C1, D1, and E1, respectively), the GFP channel is shown in white. Laser and microscope settings were constant for each marker line. Scale bars represent 100 ìm in overviews and 50 ìm in enlargements. (F) The in silico root describes the epidermis ([ep]; blue), cortex ([c]; green), endodermis ([en]; yellow), pericycle ([p]; orange), and vasculature ([v]; red). QC (grey) and columella cells (cyan) are only in the distal MZ. Scale bars represent 100 ìm. Model cell types are endowed with specific PIN topologies and strengths, which vary by zone. Differences between zones are indicated by changes in color tone. Red indicates strong PIN expression, blue weak. Typical cell lengths vary between zones, as indicated. Cell widths vary between tissue types and are kept constant through the zones. Parameter values are given in Protocol S1: Tables S1–S3 and Text S1.
