# Supplementary material for: Root System Architecture from Coupling Cell Shape to Auxin Transport
Source: PLoS Biol. 2008 Dec 16;6(12):e307. doi: 10.1371/journal.pbio.0060307 (PMC2602721; doi:10.1371/journal.pbio.0060307)
Supplement: Supplementary file 1 [file pbio.0060307.sd001.pdf]

Protocol S1 of

Root System Architecture from Coupling Cell Shape to Auxin Transport

Marta Laskowski[1,3,4], Verônica A. Grieneisen[2,3], Hugo Hofhuis[1,3], Colette A. ten Hove[1], Paulien Hogeweg[2], Athanasius F. M. Marée[2,*], Ben Scheres[1,*]

October 26, 2008

[1]Molecular Genetics Group, Dept. of Biology, Utrecht University, Padualaan 8, 3584 CH Utrecht, The Netherlands.
[2]Theoretical Biology Group, Dept. of Biology, Utrecht University, Padualaan 8, 3584 CH Utrecht, The Netherlands.
[3]These authors contributed equally to this work.
[4]Current address: Dept. of Biology, Oberlin College, Oberlin, OH, USA.
[*]Corresponding authors.

Text S1

A model of the Arabidopsis root: modeling auxin dynamics

Auxin transport is simulated on a two-dimensional (2D) grid representing a cross-section through the root, in which the bilateral symmetry of the root across the xylem axis is captured. Each cell consists of multiple grid points, such that auxin concentrations may vary within cells. Cell width differs depending on cell type: epidermal cells are $17\,\mu$m, cortical $20\,\mu$m, endodermal $12\,\mu$m, and vascular and pericycle cells $5\,\mu$m wide. Cell length varies depending on the zone in which the cell is located: cells in the MZ are $16\,\mu$m, in the EZ $60\,\mu$m and in the DZ $100\,\mu$m long. The cell wall is described as a separate entity, one grid point ($1\,\mu$m) wide (sensitivity towards different cell wall widths is explored in Fig. S5).

Auxin flow through a cell requires influx, bringing auxin into the cell across the membrane, diffusion within the cell, and efflux, allowing auxin to cross the membrane and enter the cell wall space. Once in the cell wall space, auxin can freely diffuse until it reaches a membrane. In the basic model, influx is given by a constant permeability value; P_{in}, in units of $\frac{\mu\text{m}}{s}$. In the extended model presented in Fig. 4, AUX1-mediated auxin influx is allowed to vary (see section below for details on how P_{in} is calculated). Auxin efflux permeability is assigned a strong ($P_{e_{SPIN}}$), weak ($P_{e_{WPIN}}$), or background ($P_{e_{bg}}$) value. The choice of parameter was determined by visual inspection of images showing the location and intensity of fluorescent PIN fusion proteins (see Fig. 2). Background efflux permeability $P_{e_{bg}}$ is fixed at 5% of the permeability resulting from high intensity PIN expression. All parameters are shown in Table S1 and S2.

Diffusion of auxin takes place within cells and cell walls and is given by $\vec{J} = -D\vec{\nabla}C$, where D corresponds to the diffusion constant, given in units of $\frac{\mu\text{m}^2}{\text{s}}$ (note that permeability values

Table S1: General parameters for auxin dynamics.
Parameter values used in all simulations of the main text (default parameters).

symbol	description	unit	value
Δt	time step	s (seconds)	0.1
Δx	space step	μm (microns)	1
D	auxin diffusion constant	$\mu m^2/s$	300
P_i	influx auxin permeability	$\mu m/s$	20
$P_{e_{bg}}$	background PIN efflux permeability	$\mu m/s$	1
$P_{e_{SPIN}}$	permeability due to **strong** PIN expression	$\mu m/s$	20
$P_{e_{WPIN}}$	permeability due to **weak** PIN expression	$\mu m/s$	5
δ_{decay}	auxin decay	s^{-1}	5×10^{-6}

Table S2: Additional parameters for specific simulations.
For AUX1 regulation through auxin, additional parameters were used. For the immersion in auxin simulation, a peripheral influx was added to describe auxin entering the tissue from all sides.

symbol	description	unit	value	simulations
β	half-max auxin concentration (for AUX1 response in DZ)	a.u.	17	Fig. 3F–I,K–N Movie S3,S4
n	hill-coefficient (for AUX1 response in DZ)	-	4<br>3	Fig. 3F–I,K–N Movie S3,S4<br>Movie S6
ΔT_{AUX1}	auxin time integration (for AUX1 response in DZ)	s	600	Fig. 3F–I,K–N Movie S3,S4,S6
l	peripheral influx (immersion in auxin)	$\frac{\text{a.u.}}{[s][\mu m]}$	0.01	Fig. S13

carry the unit of $\frac{\mu m}{s}$), and C corresponds to the auxin concentration, given in arbitrary units [a.u.]. Diffusion is not permitted across cell membranes (i.e. no flux boundary conditions). The flux due to auxin permeability across a membrane can be written as

$$\vec{J} = \begin{cases} -(P_{e_{SPIN}}\hat{\mathbf{n}})C_{in} + (P_{in}\hat{\mathbf{n}})C_{out} & \text{if PINs are expressed strongly,} \\ -(P_{e_{WPIN}}\hat{\mathbf{n}})C_{in} + (P_{in}\hat{\mathbf{n}})C_{out} & \text{if PINs are expressed weakly,} \\ -(P_{e_{bg}}\hat{\mathbf{n}})C_{in} + (P_{in}\hat{\mathbf{n}})C_{out} & \text{if only background efflux takes place,} \end{cases} \tag{1}$$

where $\hat{\mathbf{n}}$ is the inward directed unit vector, perpendicular to the membrane; C_{in} represents the auxin concentration in the cytosol at the grid point bordering the cell membrane; and C_{out} represents the auxin concentration in the cell wall grid point immediately adjacent to the cell membrane. Simulations of auxin dynamics are performed by concurrently solving for the diffusion, permeability and decay of auxin, using an Alternating Direction Implicit (ADI) method [1]. For the diverse control simulations performed, different tissue segments have been simulated, some up to 3500x4000 pixels (Fig. S11C).

Table S3: Parameter sweeps performed for control simulations.
Special simulations in which default parameters have been modified, for parameter robustness studies, are listed below with corresponding parameter values.

Δx in μm	simulations
2	Fig. S5C
1	Fig. S5D
0.5	Fig. S5E
0.2	Fig. S5F

D in $\mu m^2/s$	simulations
600	Fig. S3A1,B1,C1
100	Fig. S3A3,B3
50	Fig. S3A4,B4

D_{cw} in $\mu m^2/s$	simulations
40	Fig. S3C1,D1, Fig. S4B2
20	Fig. S3C2,D2

Permeability in $\mu m/s$	simulations
all 10 times lower:<br>$P_i = 2$<br>$P_{e_{bg}} = 0.1$<br>$P_{e_{SPIN}} = 2$<br>$P_{e_{WPIN}} = 0.5$	Fig. S4B
all 100 times lower:<br>$P_i = 0.2$<br>$P_{e_{bg}} = 0.01$<br>$P_{e_{SPIN}} = 0.2$<br>$P_{e_{WPIN}} = 0.05$	Fig. S4C

Bending the *in silico* root

Bending the *in silico* roots consists of transforming cell shapes at the region selected, and rotating the linked distal straight segment of the root. Thus, pixels representing cells, cell walls and their corresponding auxin concentrations are mapped from their initial state A to a transformed state B. The parameters that fully describe the transformation are:

$y1, y2$ — Longitudinal (i.e. axial direction) begin and end points of the bent segment.

c — Curvature of the region to be bent, where $c = \frac{1}{r}$, in which r is the radius of curvature.

x_0 — Latitudinal (i.e. transversal) position within the root that determines the reference line in relation to which expansion or compression occurs. For example, if x_0 is exactly in the middle of the root, then the outer half of the root will have increased cell lengths, and the inner half decreased cell lengths.

These are all the parameters used to calculate the mathematical projection of the grid describing the root before bending to one describing the bent root. We applied four corrections during this transformation. First, cell shapes change during deformation, which means that some grid points occupy multiple grid points after the bending, while other grid points disappear. At the moment of bending, auxin levels are scaled relative to the change in the area of the cells, such that the total amount of auxin per cell remains the same. This scaling generates a strong transitory bias towards the *inner* side of the curve, this opposing bias (i.e. in opposite direction to the one we observe generated through the mechanism proposed here) disappears within a minute. Implications of this transformation for 3D cell volumes as well as robustness towards volume conservation are discussed and shown in Fig. S7. Second, the fact that grid points are not conserved during bending, can cause local disappearance or doubling of the cell wall, which is unrealistic. Therefore, during the transformation, cell wall widths are held constant. (See Fig. S5 for further discussion.) Third, because the bending causes changes in the membrane length, permeability values are scaled to reflect a constant amount of PIN expression per cell. (The implications as well as a relaxation of this assumption are discussed in Fig. S6.) Fourth, in most simulations a strong bend is used, with a 180° curve over 3 cells. In such a case, when x_0 is positioned exactly in the middle of the root, the relative size differences between the cells become very large. We therefore used a 37.5 μm (1/4th root width) offset towards the inner curve for the value of x_0, which leads to a more than 50% reduction in the ratio between the outer and inner boundary length, compared to using a value of x_0 exactly at the middle of the root. (For further discussion of auxin increases and biases in weakly curved roots, see Fig. S10 and Fig. S11.)

AUX1 upregulation

The feedforward regulation of AUX1 by auxin is implemented using a moving average of the intracellular auxin concentration. The variable $C_i(t)$ gives the mean auxin concentration within cell i, integrated over a time window ΔT_{AUX1}, typically 10 min. Time windows between 5 and 50 minutes reproduced the basic patterns reported here (data not shown). The level of AUX1 expression, $A_i(t)$, of cell i at a given moment t, is given through the sigmoidal relation

$$A_i = \frac{C_i^n}{\beta^n + C_i^n},$$

where β is the auxin concentration at which AUX1 expression reaches half its maximum response, and n determines the steepness of the response. The AUX1 expression enhances auxin influx along all sides of the cell. We use the same functional response with the same parameters, for all cell types within the Differentiation Zone (DZ), both within and outside of the bent region. Expression of the AUX1 auxin importer changes dynamically, as a consequence of the absolute levels of auxin within the same cell. Note that only local information is used: relative differences in auxin levels between neighboring cells do not play a role. Changes in auxin concentrations result from modifications of local fluxes, which depend in part on the influx rates along the membrane of the cell and its close neighbors. At each position along the membrane of a cell i, the influx is given by

$$J_{in} = P_{in}C_{out} + A_iC_{out},$$

where P_{in} is the default influx permeability, A_i the permeability due to AUX1 expression, and C_{out} the local concentration within the cell wall, just outside the membrane. The efflux permeability (given by P_{bg} and $P_{e_{S,WPIN}}$) are unaffected during this process. The total efflux, however,

does increase, due to the increasing internal auxin concentrations, as the efflux is given by $J_{out} = P_{out}C_{in}$, where P_{out} is either P_{bg} or $P_{e_{S,WPIN}}$, and C_{in} is the internal cellular auxin concentration close to the membrane point of consideration. The auxin-AUX1 feedforward loop is not inherently unstable, because AUX1 upregulation reduces the reflux, and thereby the total auxin throughput (see Movie S4).

Supporting Figures

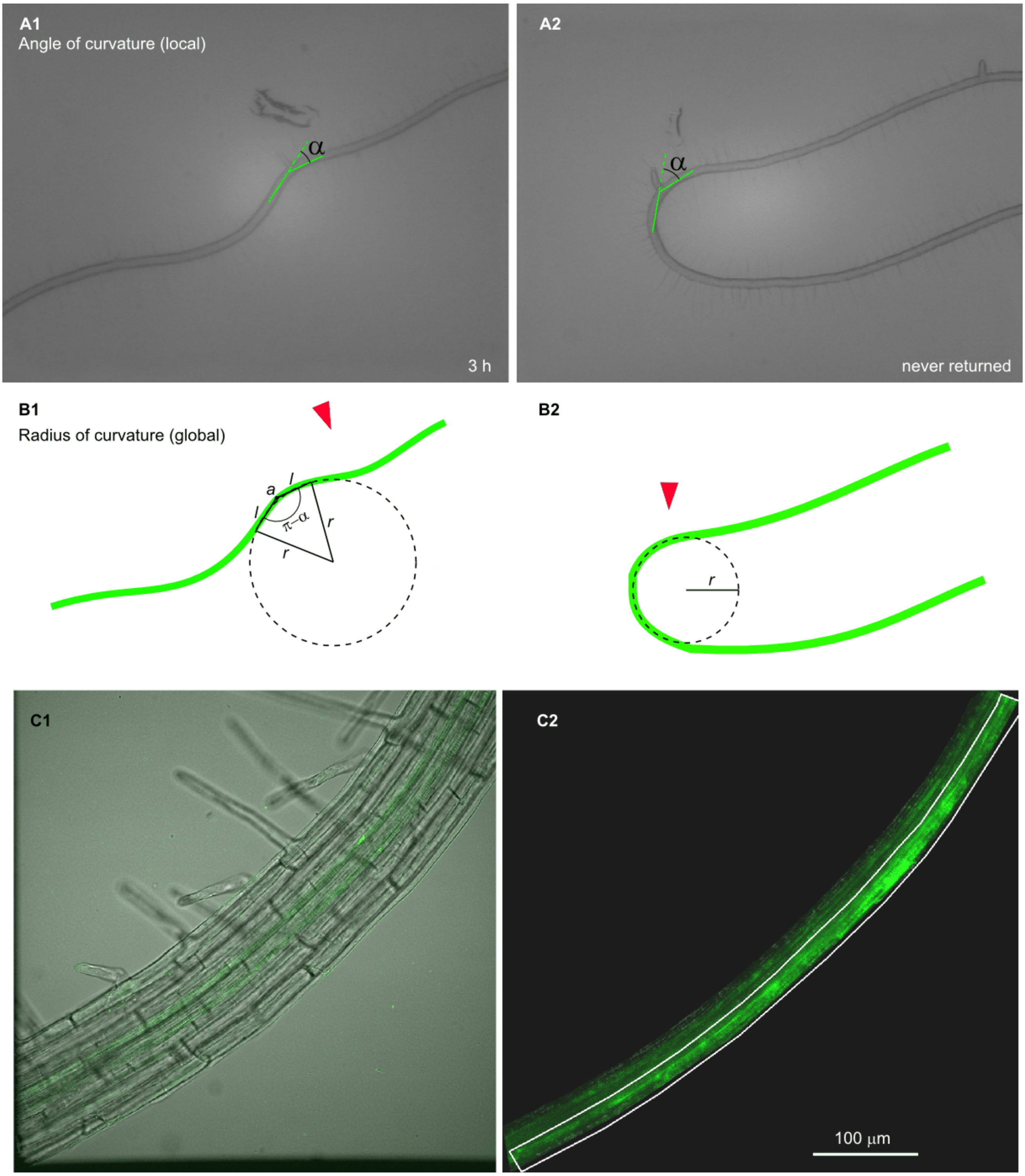

Figure S1

Figure S1: Measuring local curvatures.
(A) The 'angle of curvature' presented in Fig. 1E was determined by drawing two line segments (of length l) that join at the point where the curvature reaches a local maximum. Line segments were positioned with their end points in the middle of the root's width. Examples are shown for a root that was inverted for a period of 3 h (A1), and for a root permanently left in inverted position (A2). This angle of curvature is a local property of the curve at the position a, however, it can be related to the radius of curvature, as illustrated in (B). (B) Schematics showing how the angle of curvature (above and Fig. 1E), are related to the radius of curvature (Fig. S9). This conversion can be made using the formula

$$r = \frac{(1/2)l}{\cos(\frac{\pi-\alpha_{rad}}{2})}, \tag{2}$$

where l is the length of the line segments utilized and α_{rad} is the measured local curvature angle, expressed in radians. **(C)** Overview of the region in which fluorescence was measured to collect the data for Fig. 1G. Scale bar is 100 μm. (C1) Image showing fluorescence concentrated in the vasculature. (C2) The vascular region is divided into two sections: inner and outer. The outer section is indicated by the white box.

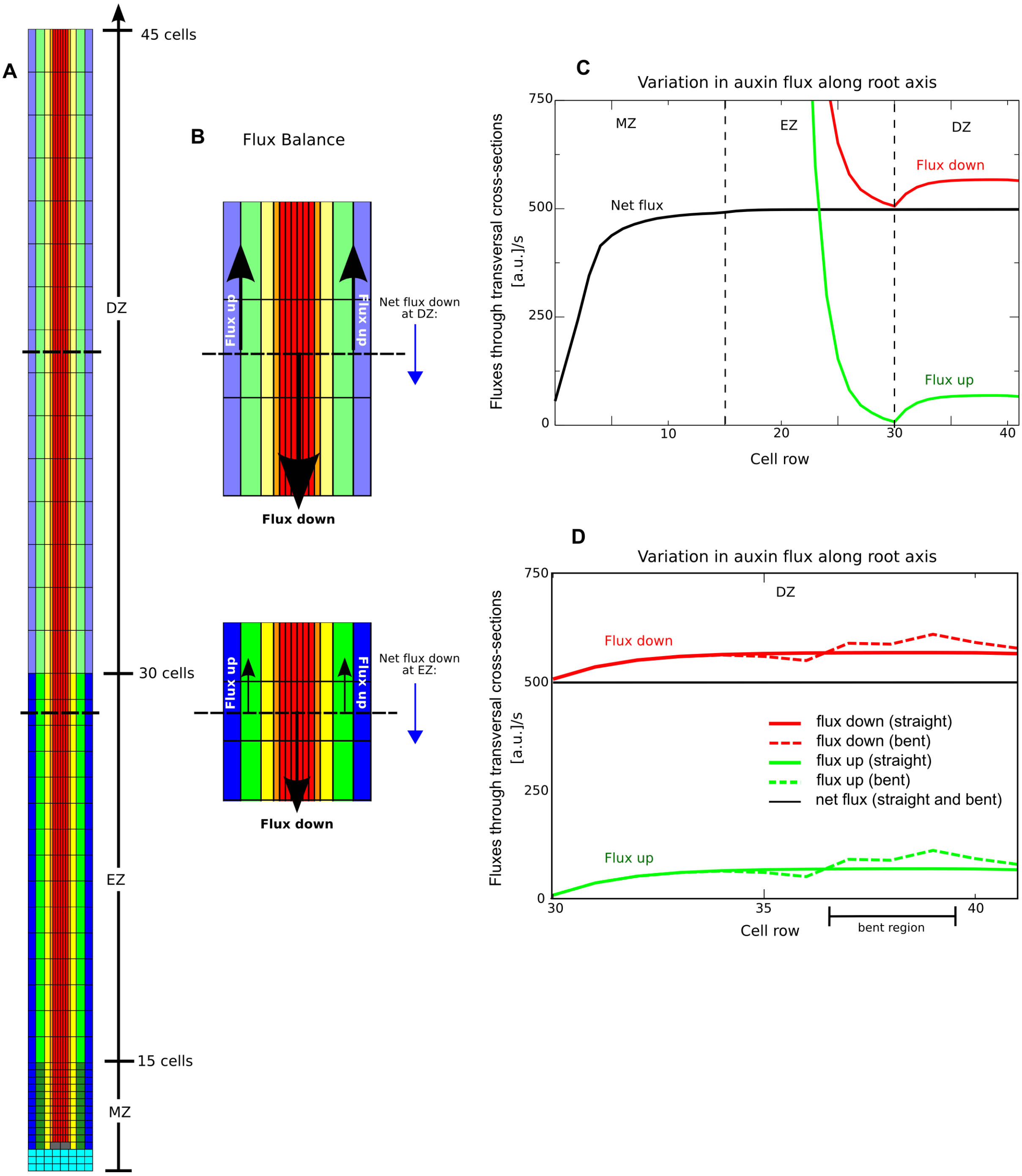
A
45 cells
DZ
30 cells
EZ
15 cells
MZ
B
Flux Balance
Flux up
Flux up
Net flux down at DZ:
Flux down
Flux up
Flux up
Net flux down at EZ:
Flux down
C
Variation in auxin flux along root axis
Fluxes through transversal cross-sections [a.u.]/s
MZ
EZ
DZ
Net flux
Flux down
Flux up
Cell row
D
Variation in auxin flux along root axis
Fluxes through transversal cross-sections [a.u.]/s
DZ
Flux down
Flux up
flux down (straight)
flux down (bent)
flux up (straight)
flux up (bent)
net flux (straight and bent)
Cell row
bent region

Figure S2

Figure S2: Auxin fluxes through the root.
Here, we show the net vertical flux through transversal cross-sections of the root tissue as a function of axial root position. The apical and basal flux components – which sum up to the net vertical flux – are shown alongside. From the alterations in magnitude of the apical and basal fluxes, lateral flows can be inferred. This analysis allows us to elucidate quantitatively the influence of root zonation on auxin flux patterns. **(A)** Model root layout. The first 15 cell rows comprise the Meristem Zone (MZ), the next 15 cell rows are the Elongation Zone (EZ), and the most proximal cells form the Differentiation Zone (DZ). PIN distributions are as given in Fig. 2. **(B)** At equilibrium, the net downward flux is equal for any cross-section through the root that is sufficiently far away from the distal MZ. This is a consequence of auxin predominantly accumulating in the distal MZ [2], due to which auxin decay outside the distal MZ only plays a marginal role. Thus, the net flux passing through the two dotted lines in (A) (see (B) for enlargements), should be almost equal. **(C)** Fluxes across transversal cross-sections at the center of mass of each cell row (such as the dotted lines in (B)) in a steady state auxin profile. The graph shows clear differences between distal MZ/EZ, proximal EZ, and DZ fluxes. In red, the total downward flux; in green, the total upward flux; and in black, their sum, i.e. the net downward flux is shown. Fluxes are exceedingly high in the distal MZ (see Fig. 3 for concentration values), thereby leaving the graph; here, however, we specifically focus on the alterations at the EZ/DZ boundary, to which the y-axis has been adjusted to. Note that the net flux remains constant until the very distal region. Only at the root tip, with its huge auxin accumulation, does auxin breakdown start to significantly influence net flux patterns. Towards the proximal end of the EZ, there is a clear dip in the upward and downward fluxes, which is rapidly re-established after entering the DZ. This increase reflects the presence of lateral reflux. **(D)** Graph focusing on the proximal portion of the described root, comparing total upward and downward fluxes within a bent and an unbent root. The black line indicates the curved region. Bending causes marked effects in both flux components: both upward and downward fluxes significantly increase at the bend, but decrease in the region just distal to the bend. The net flux, however, remains constant, as it should be in an equilibrium situation (neglecting breakdown). This quantitatively illustrates the importance of the lateral flux components that arise due to bending, and explains the (initially counter-intuitive) observation that lower PIN expression in the DZ zone actually accounts for higher flux patterns. It also illustrates how being able to generate a reflux loop (auxin exiting the vasculature and reentering it proximally from the external files) is essential to feedforward the initial cell-shape induced changes in the auxin levels.

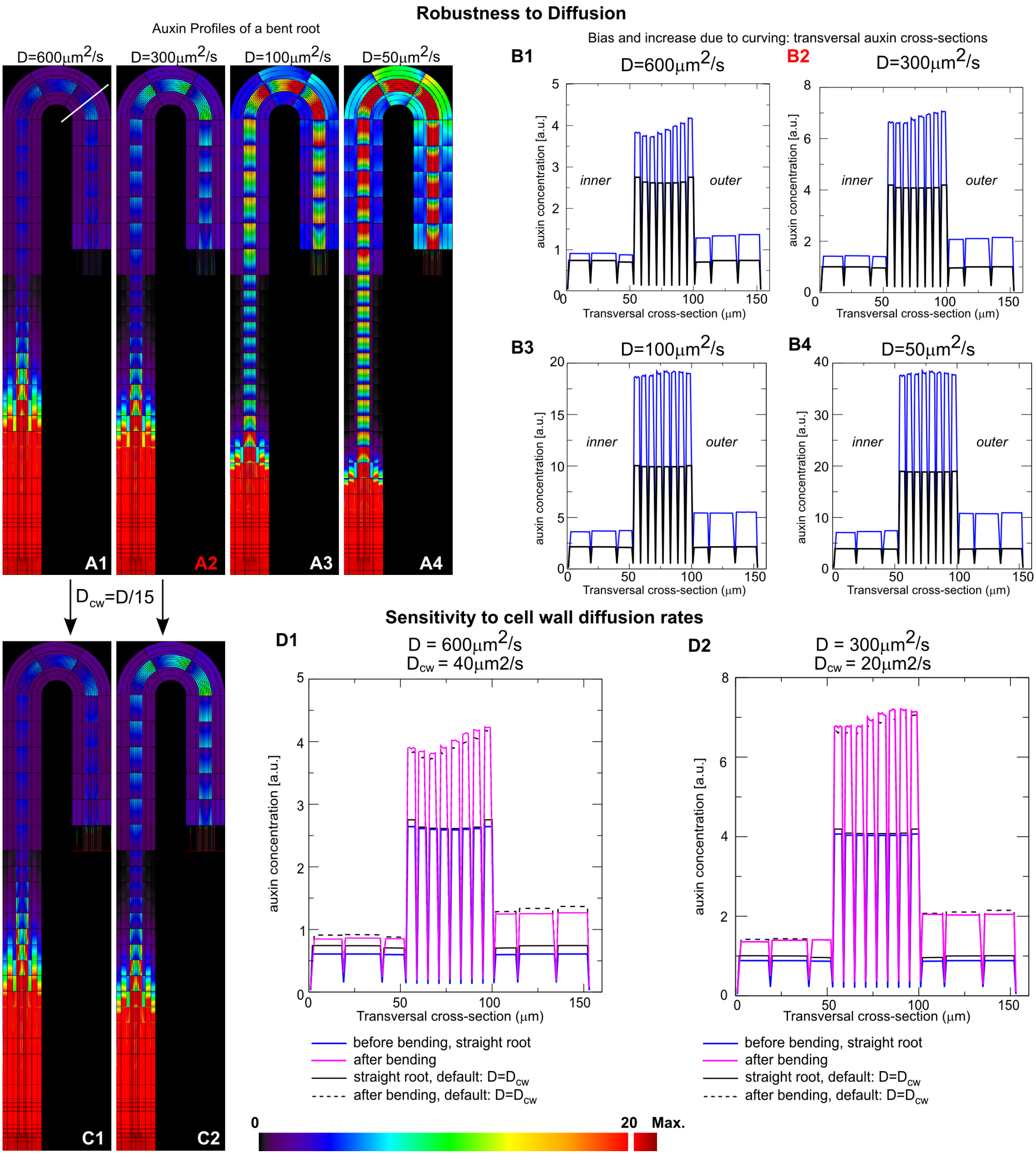

Robustness to Diffusion
Auxin Profiles of a bent root
D=600μm2/s
D=300μm2/s
D=100μm2/s
D=50μm2/s
A1
A2
A3
A4
Bias and increase due to curving: transversal auxin cross-sections
B1
D=600μm2/s
B2
D=300μm2/s
B3
D=100μm2/s
B4
D=50μm2/s
inner
outer
auxin concentration [a.u.]
Transversal cross-section (μm)
Dcw=D/15
C1
C2
Sensitivity to cell wall diffusion rates
D1
D = 600μm2/s
Dcw = 40μm2/s
D2
D = 300μm2/s
Dcw = 20μm2/s
before bending, straight root
after bending
straight root, default: D=Dcw
after bending, default: D=Dcw
0
20
Max.

Figure S3

Figure S3: Robustness towards auxin diffusion constants.
Neither uniformly changing auxin diffusion constants by more than an order magnitude nor decreasing auxin diffusion constants within the cell wall prevents auxin from accumulating in the vascular cells on the outside of the bend. **(A)** Auxin profiles in model roots with various auxin diffusion constants. **(B)** Profiles of transversal cross-sections (blue) through the center of the most proximal cell at the bend (indicated by a white line in A1), compared with the profile at that location before bending (black). B2 (red letter) is the default situation. (A1, B1) Diffusion constant $D = 600\,\mu\text{m}^2/\text{s}$; (A2,B2) diffusion constant corresponding to the default values used for the simulations in the main text, i.e. $D = 300\,\mu\text{m}^2/\text{s}$; (A3, B3) $D = 100\,\mu\text{m}^2/\text{s}$; and (A4, B4) $D = 50\,\mu\text{m}^2/\text{s}$. The curvature-induced increase in auxin concentrations observed in both vasculature and the outer external cell files at the bend, as well as the outside/inside bias is robust towards these diffusion constants. Note that higher diffusion constants do have the tendency to flatten out internal cellular gradients (A1), but the outer bias is still preserved. Lowering the diffusion constants only enhances the increase in auxin due to bending, as can clearly be seen by the dramatic increase of auxin (more than 100% rise in pericycle cells) in (A4, B4).

(C,D) Experiments performed by Kramer et al. [3] have led to estimates that the diffusion of auxin in the cell wall is 15 times lower than in the cytosol. Throughout the main text, we have not included these differences in the model. Simulations in which a 15-fold decrease in cell wall diffusion is taken into account (using as a basis the parameter values of A1 and A2), reveal only minor effects in the auxin profiles. **(C)** Auxin concentration profiles when cytosolic diffusion constants are $600\,\mu\text{m}^2/\text{s}$ (C1) and $300\,\mu\text{m}^2/\text{s}$ (C2), with corresponding cell wall diffusion constants of $40\,\mu\text{m}^2/\text{s}$ and $20\,\mu\text{m}^2/\text{s}$. Color bar indicates auxin concentrations. **(D)** Transversal cross-section comparing auxin concentration profiles at the site of the bend for the situation in which diffusion is 15 times lower in the cell wall (solid blue line for straight and solid magenta line for bent situation) as well as when it is considered equal in the cell wall and cytosol (black lines; solid for straight and dotted for bent situation).

Robustness of bending effects towards permeability rates

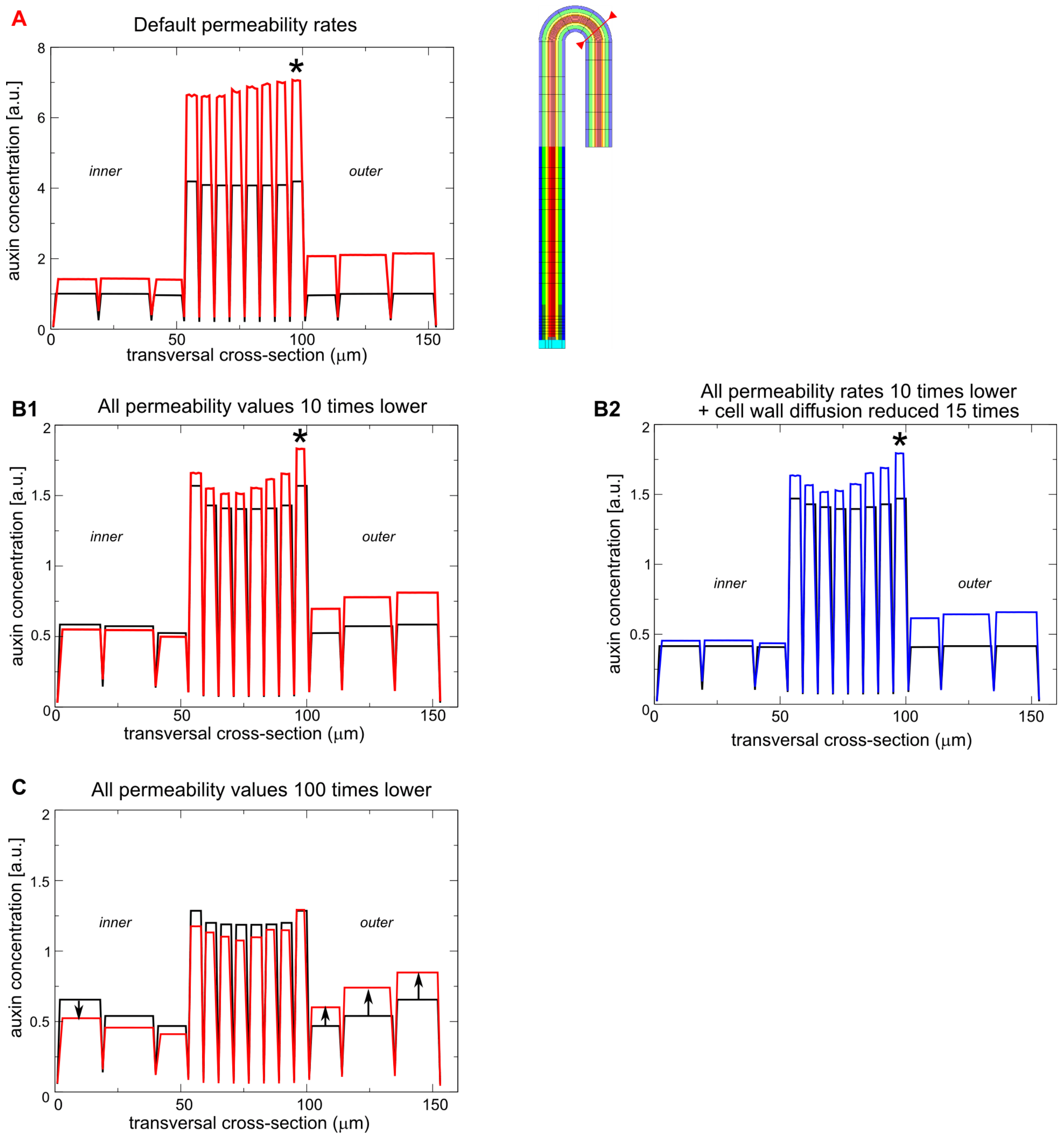

Figure S4

Figure S4: Sensitivity towards permeability parameters.
Exact values of PIN-dependent auxin permeability through a membrane in which PIN expression levels have been experimentally verified, have yet to be established. The default values used here are based on Grieneisen et al. [2] and references therein. Increasing these default values causes internal cellular gradients to become steeper, enhancing the effect of curvature (data not shown) while decreasing the default values can diminish the response. Delbarre et al. [4] performed measurements on protoplasts, and estimated efflux permeability values that are lower than the ones used here. These values have served as reference for parameter choices used in other modeling studies [5–7] (leading to ~10 times lower PIN-mediated permeability value than our default values used here). However, the measurements on the protoplasts lack evidence for strong PIN expression, and might present an underestimation of the PIN-mediated permeability. Given this uncertainty in actual permeability values, we investigated the effect of lowering our default values (which alternatively might be an overestimation of the permeability values), one or two orders of magnitude. **(A–C)** Transversal auxin concentration profiles for a root before (black line) and after bending (red/blue line). **(A)** Default permeability values yield the typical result at the bend: an outer auxin bias, maximum auxin increase at the outer pericycle, and an overall increase in auxin in both the inner and outer cell files at the bend. **(B1)** A 10-fold decrease in permeability consistently yields the same qualitative results, with the slight modification that the inner external cell files now present a tiny drop in auxin level. **(B2)** When the parameter settings of (B) are combined with the assumption that diffusion is lower in the cell wall (a 15-fold reduction), the auxin profile remains qualitatively similar, while the auxin concentrations in the inner external cell files slightly rise again. Thus, lowering permeability values decreases the magnitude of the auxin concentrations (note the change in the scale of the y-axis between A and B), but does not alter the overall pattern (i.e. relative differences) of auxin distribution. **(C)** A 100-fold decrease in the permeability values has a more noticeable effect on the qualitative aspects of the auxin redistribution after bending: the outer bias is clearly preserved, as well as the auxin increase in the outer cell files. However, not much change can be observed in the pericycle itself, and a clear drop marks the inner external cell files. We conclude that the formation of an outer bias is very robust towards uniform changes in permeability, while the increase of auxin in the inner external cell files is less so.

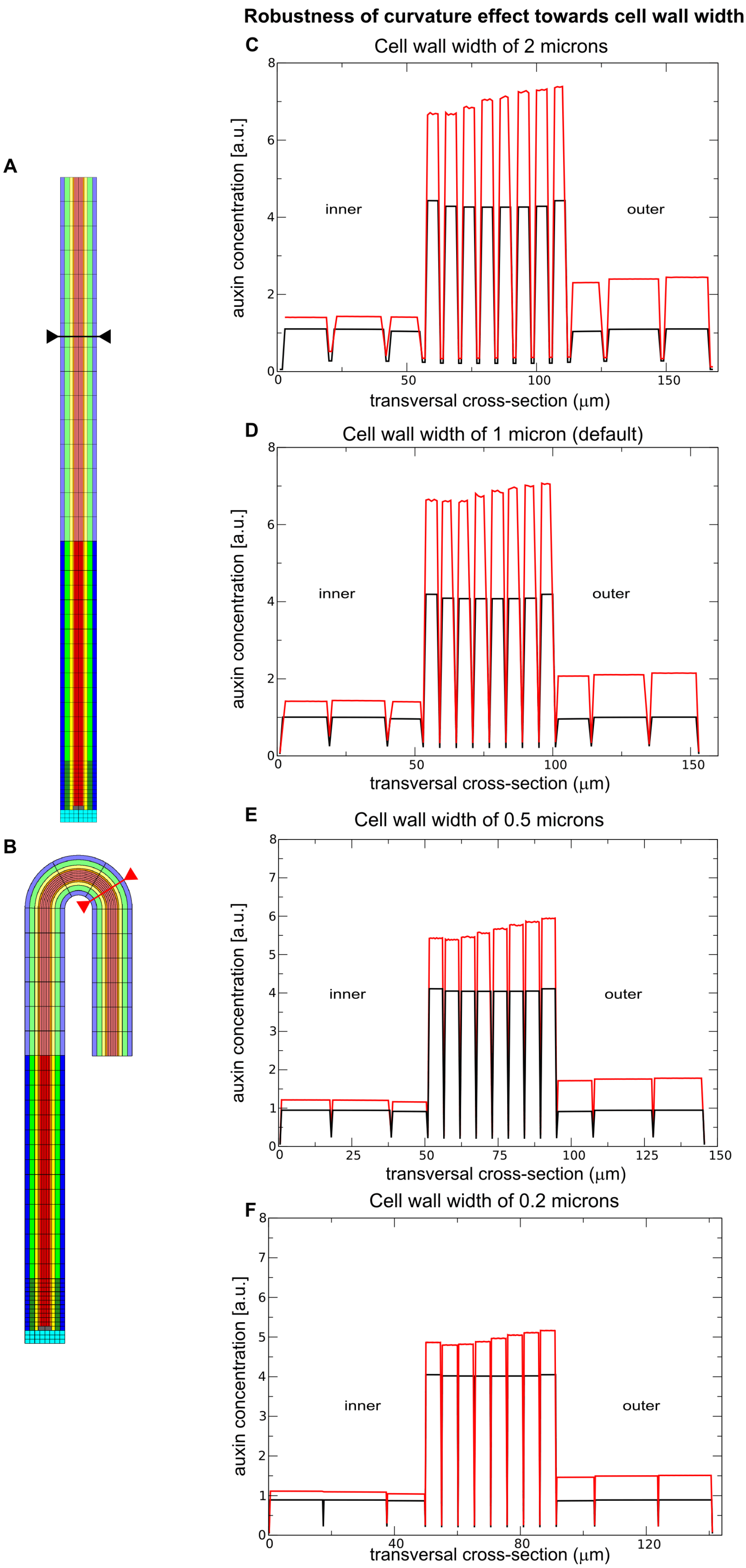
Robustness of curvature effect towards cell wall width
A
B
C
Cell wall width of 2 microns
D
Cell wall width of 1 micron (default)
E
Cell wall width of 0.5 microns
F
Cell wall width of 0.2 microns
auxin concentration [a.u.]
transversal cross-section (μm)
inner
outer

Figure S5

Figure S5: The influence of cell wall width.
Cell wall widths vary within the Arabidopsis root depending on the cell type, age, and region of the root, and possibly respond to mechanical deformations. In our model, we we used a constant cell wall width of 1 μm as a default setting in both straight and bent regions of the root. Here, we present the outcome of multiple simulations, in which the cell wall widths have been varied between 0.2 μm and 2 μm. Auxin concentrations are shown for cross-sections through the root, indicated by the black and red lines, for the straight and bent root respectively. **(A,B)** Location of the transversal cross-sections. **(C–F)** Transversal cross-sections showing auxin profiles for simulations with cell wall width set to 2 μm (C); 1 μm (D); 0.5 μm (E); and 0.1 μm (F). These results show uniform changes in cell wall width have little impact on the formation of an auxin increase and bias, with thicker cell walls presenting larger increases in auxin at the bend. Consequently, if root bending increases the cell wall width in a graded fashion, this would predict a further increase in the bias.

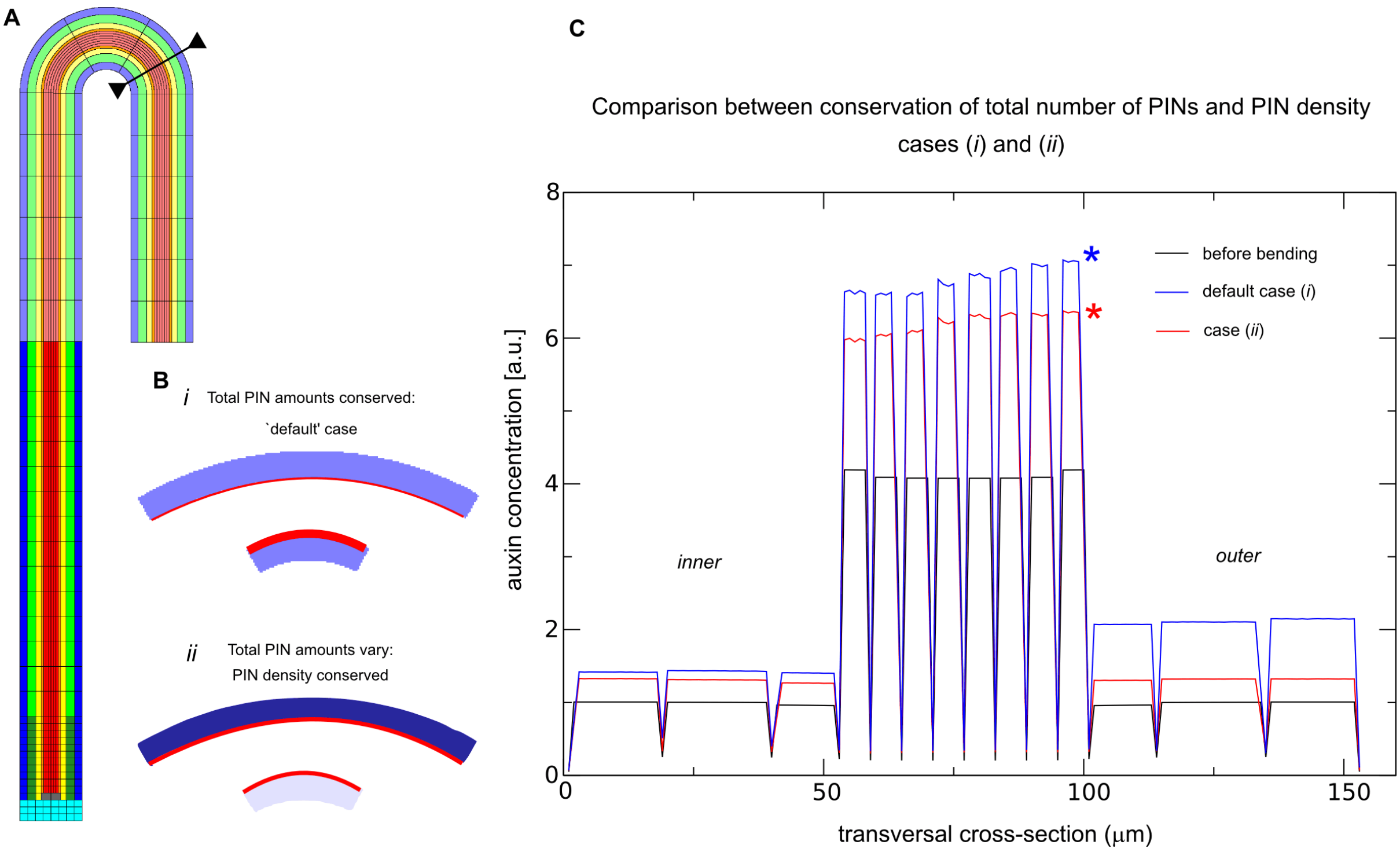

Figure S6: PIN density alterations due to cell shape change.
When bending the *in silico* root, as indicated in **(A)**, the model assumes that the total amount of PIN expression is not altered during the cell shape change. Consequently, when the length of a lateral membrane of a cell decreases, the PIN density along the membrane increases. Similarly, when perimeter increases, the PIN density is 'diluted' proportionally. This default case is schematically represented in **(B)**, case *i*, where the intensity of blue represents total PIN protein amounts, and the thickness of the red lateral line represents the membrane PIN density. Here, we consider the contrasting situation **(B)**, case *ii*, in which PIN density is held constant, allowing total PIN amounts to vary. **(C)** Comparison between transversal auxin profiles for case *i* and *ii*. The cross-sections go through the cell row at the bend indicated in (A). Both assumptions result in an increase of auxin in the vascular cells located at the bend (red and blue lines), compared to the profile before bending (black line), however, conservation of PIN density on the membrane reduces the bias between inner and outer external cell files. Note that these simulations represent extremes, while in reality the situation is most likely a combination of compensatory homeostatic control with (initially) limited amounts of PIN protein. Also, one would expect the kinetics of PIN expression readjustment after bending to play a role.

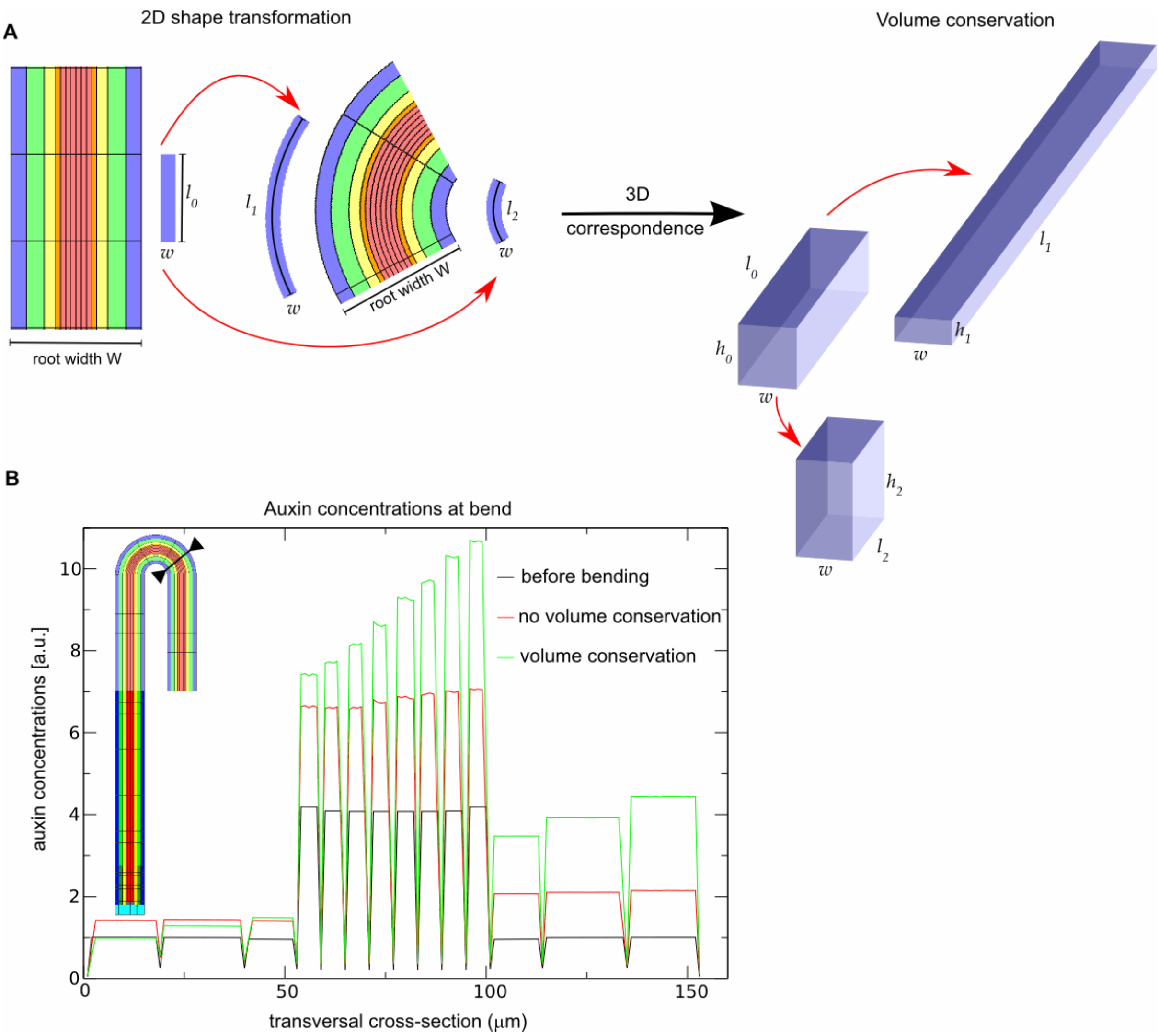

Figure S7: Cell volume conservation.
(A) In the 2D root-bending method that we employ, the 2D cross-sectional area of a cell changes when it is bent. This implies that the volume of cells change, an assumption that might or might not be realistic. To explore the possible effects of volume conservation, and to determine if the mechanism we report still holds if volume is conserved, we developed a mathematical method to adjust internal cellular auxin concentrations of the 2D cross-section, in a manner that corresponds to its 3D volume being conserved, schematically represented in **(A)**. **(B)** Transversal auxin profile, of an *in silico* root in which cell volume is allowed to change during bending (red, default case) compared to a case in which cell volume is conserved (green). The auxin profile before bending is shown in black. Note that the auxin increase and bias is much more pronounced when cell volume is conserved. Thus, the default assumption used in the main text might underrepresent the strength of the auxin bias.

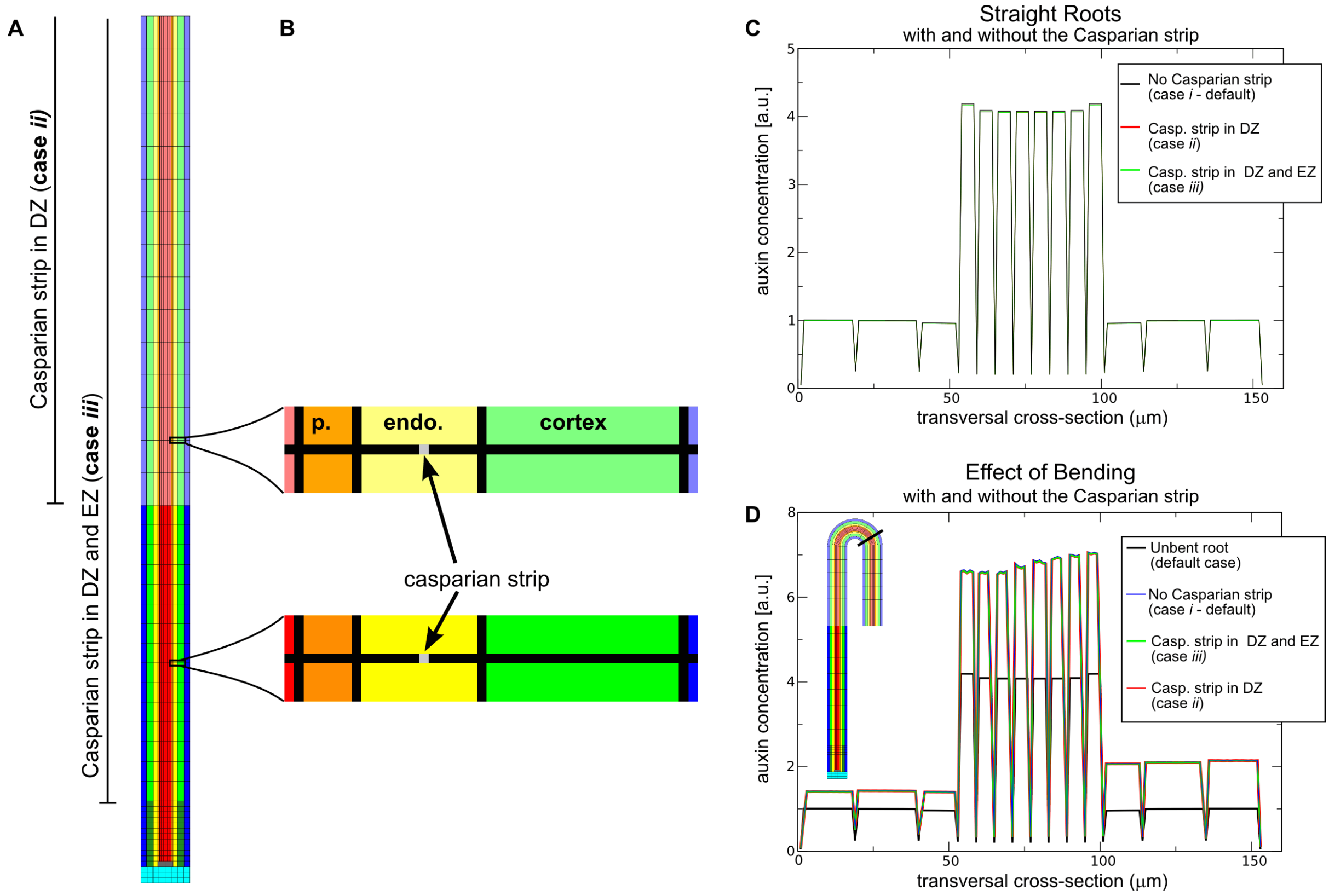

A
Casparian strip in DZ (case ii)
Casparian strip in DZ and EZ (case iii)
B
p.
endo.
cortex
casparian strip
C
Straight Roots
with and without the Casparian strip
No Casparian strip (case i - default)
Casp. strip in DZ (case ii)
Casp. strip in DZ and EZ (case iii)
auxin concentration [a.u.]
transversal cross-section (μm)
D
Effect of Bending
with and without the Casparian strip
Unbent root (default case)
No Casparian strip (case i - default)
Casp. strip in DZ and EZ (case iii)
Casp. strip in DZ (case ii)
auxin concentration [a.u.]
transversal cross-section (μm)

Figure S8

Figure S8: Casparian strip.
Introducing a Casparian strip into the *in silico* root by adding an auxin impermeable barrier to the endodermal cells does not prevent the formation of an auxin bias in the bent roots. **(A)** Layout of the model roots. In the default, case *i*, no Casparian strip is implemented; in case *ii*, a Casparian strip is located in the DZ; and in case *iii,* the Casparian strip is present in both DZ and EZ. **(B)** Inset showing the Casparian strip, implemented as an auxin-impermeable strip in the endodermal cell wall. In this cross section of the root, the strip appears as a vertical line on the apical and basal side of the endodermal cells. **(C)** Transversal auxin profiles in the DZ of straight roots, showing nearly identical outcomes for roots with and without a Casparian strip (concentration differences are <0.5%). (**D**) The presence of the Casparian strip does not substantially alter the increase in auxin at the bend, nor the bias (Black line: straight root; colored lines: bent roots). Again, the concentration differences are <0.5%). These results show that lateral auxin flux through the endodermal cells is much more important than the lateral flux through the anticlinal cell walls.

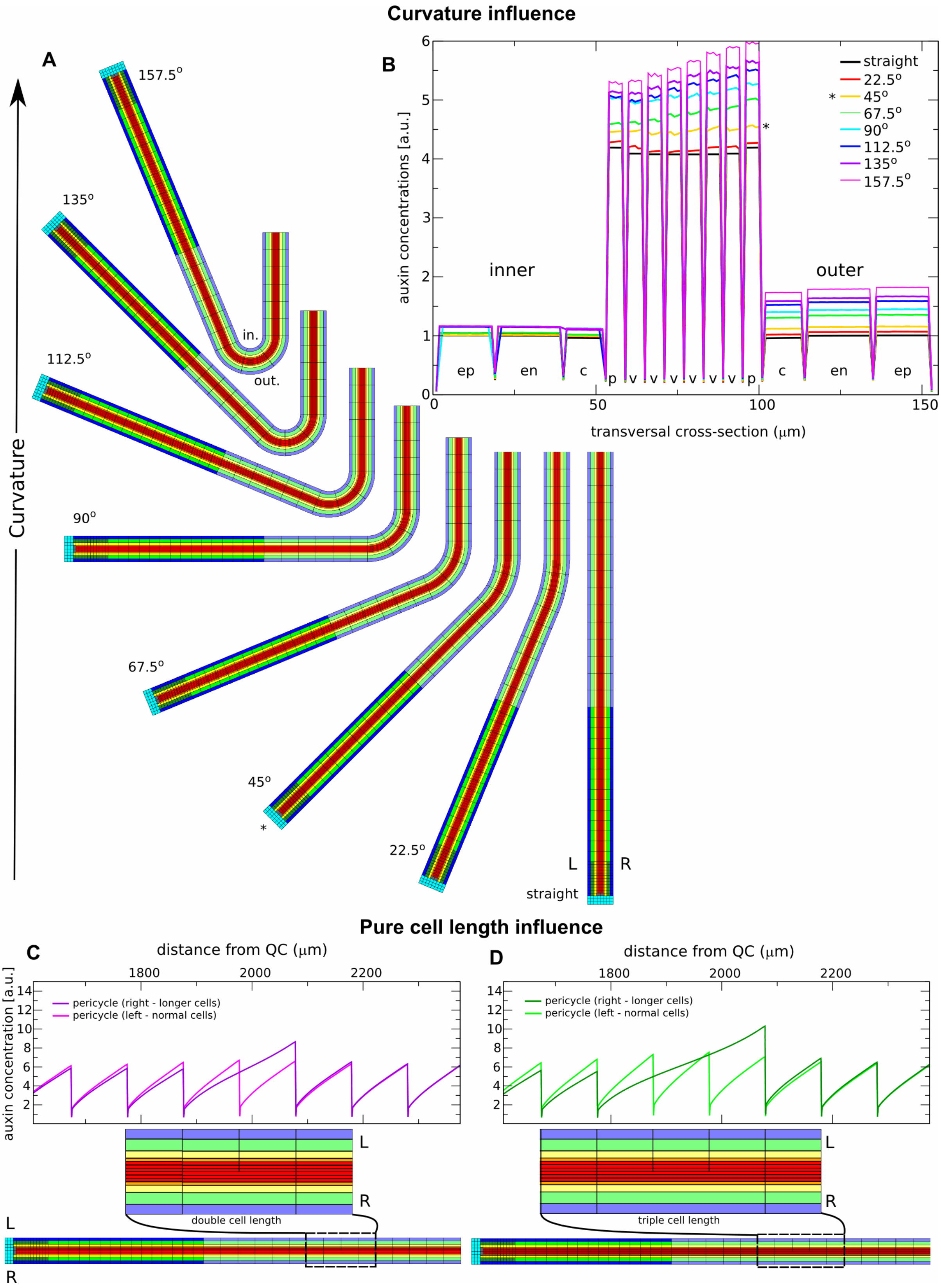

Curvature influence
A
157.5°
135°
112.5°
90°
67.5°
45°
22.5°
straight
in.
out.
L
R
Curvature
B
auxin concentrations [a.u.]
transversal cross-section (μm)
inner
outer
ep
en
c
p
v
straight
22.5°
45°
67.5°
90°
112.5°
135°
157.5°
Pure cell length influence
C
D
distance from QC (μm)
auxin concentration [a.u.]
pericycle (right - longer cells)
pericycle (left - normal cells)
double cell length
triple cell length

Figure S9

Figure S9: Curvature and cell length effects.
(A) Model root bent to different degrees, as indicated (angle formed in relation to the straight primary root axis). **(B)** Transversal cross-sections through the center of the most proximal cell at the bend. At the bend, an overall increase in auxin concentrations is observed in both the vasculature and the outer external cell files. The outside/inside bias correlates with the amount of curvature, becoming evident at angles greater than 45°. **(C,D)** Steady state auxin profiles (here plotted along a longitudinal cross-section through both the left and right pericycle cell file), in roots containing a zone with cells along the right-hand-side that are twice (C) or three times (D) as large.

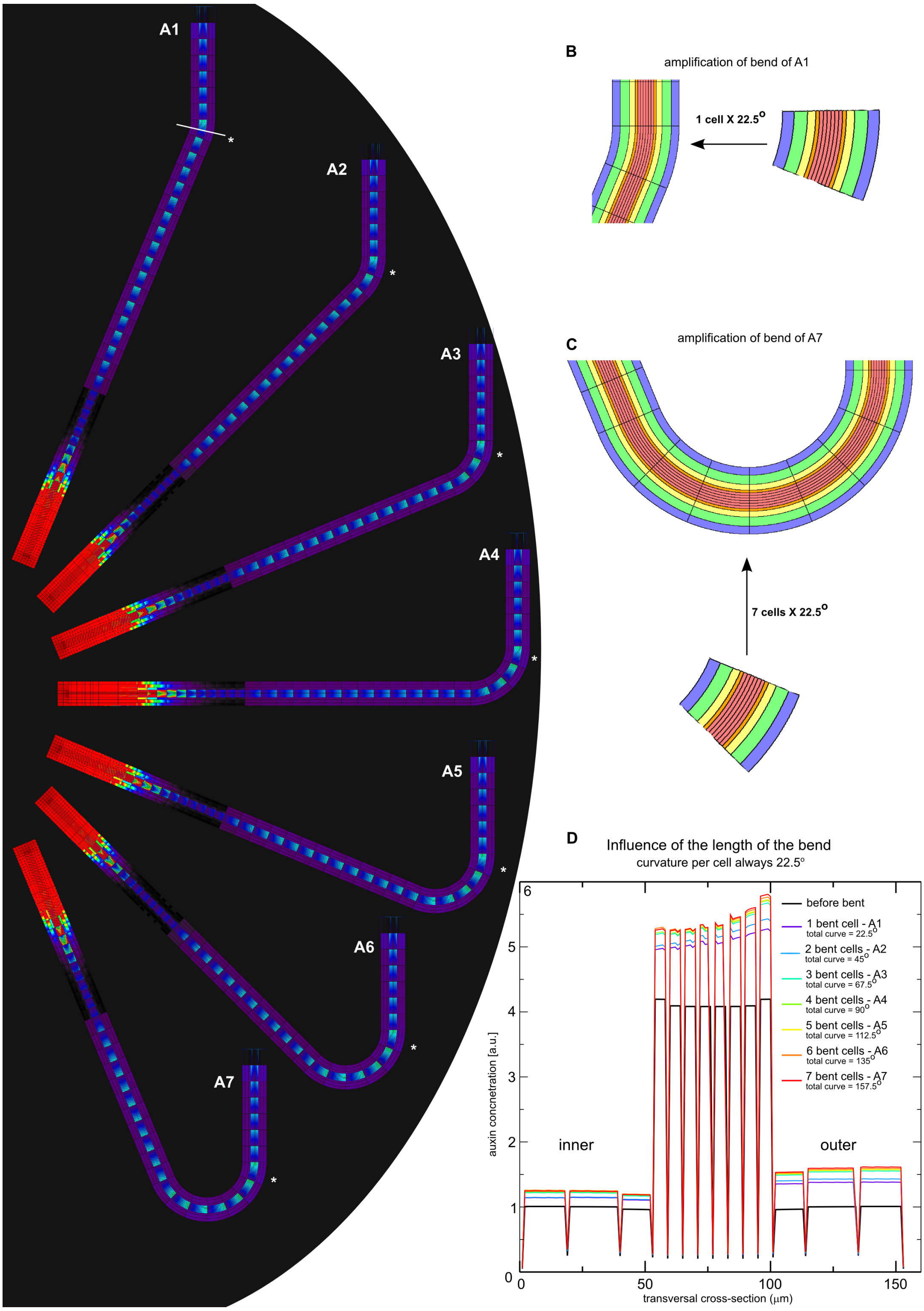
A1
A2
A3
A4
A5
A6
A7
B
amplification of bend of A1
1 cell X 22.5°
C
amplification of bend of A7
7 cells X 22.5°
D
Influence of the length of the bend
curvature per cell always 22.5°
before bent
1 bent cell - A1
total curve = 22.5°
2 bent cells - A2
total curve = 45°
3 bent cells - A3
total curve = 67.5°
4 bent cells - A4
total curve = 90°
5 bent cells - A5
total curve = 112.5°
6 bent cells - A6
total curve = 135°
7 bent cells - A7
total curve = 157.5°
inner
outer
auxin concnetration [a.u.]
transversal cross-section (μm)

Figure S10

Figure S10: Effect of increasing the length of the bent region.
To assess the effect of auxin refluxes at the bend (Fig. S2D) on the rising auxin concentrations and bias, different simulations are compared in which the same curvature is applied to the root, but the length of the curved region is varied. Simulations in which the curve is composed of $0-7$ cells, each cell of which has an angle of curvature of $22.5°$ degrees, show that the magnitude of the auxin accumulation at the bed is directly influenced by the length of the curve. **(A)** Auxin profiles for different curved arc lengths. Color bar (not shown) as in Fig. S3. 1 bent cell generates an angle between the main root axis and the distal region of $\beta = 22.5°$ (A1); 2 bent cells, $\beta = 45°$ (A2); 3 bent cells, $\beta = 67.5°$ (A3); 4 bent cells, $\beta = 90°$ (A4); 5 bent cells, $\beta = 112°$ (A5); 6 bent cells,$\beta = 135°$ (A6); and 7 bent cells, $\beta = 157.5°$ (A7). **(B–C)** Amplified view of the region of curvature for (A1) and (A2), respectively. **(D)** Transversal auxin profiles for the 8 simulations. The length of the bent region strongly influences the magnitude of the auxin increase of the outer pericycle. These results further show that cell shape differences, not only act local but can cooperate globally to elevate auxin accumulation.

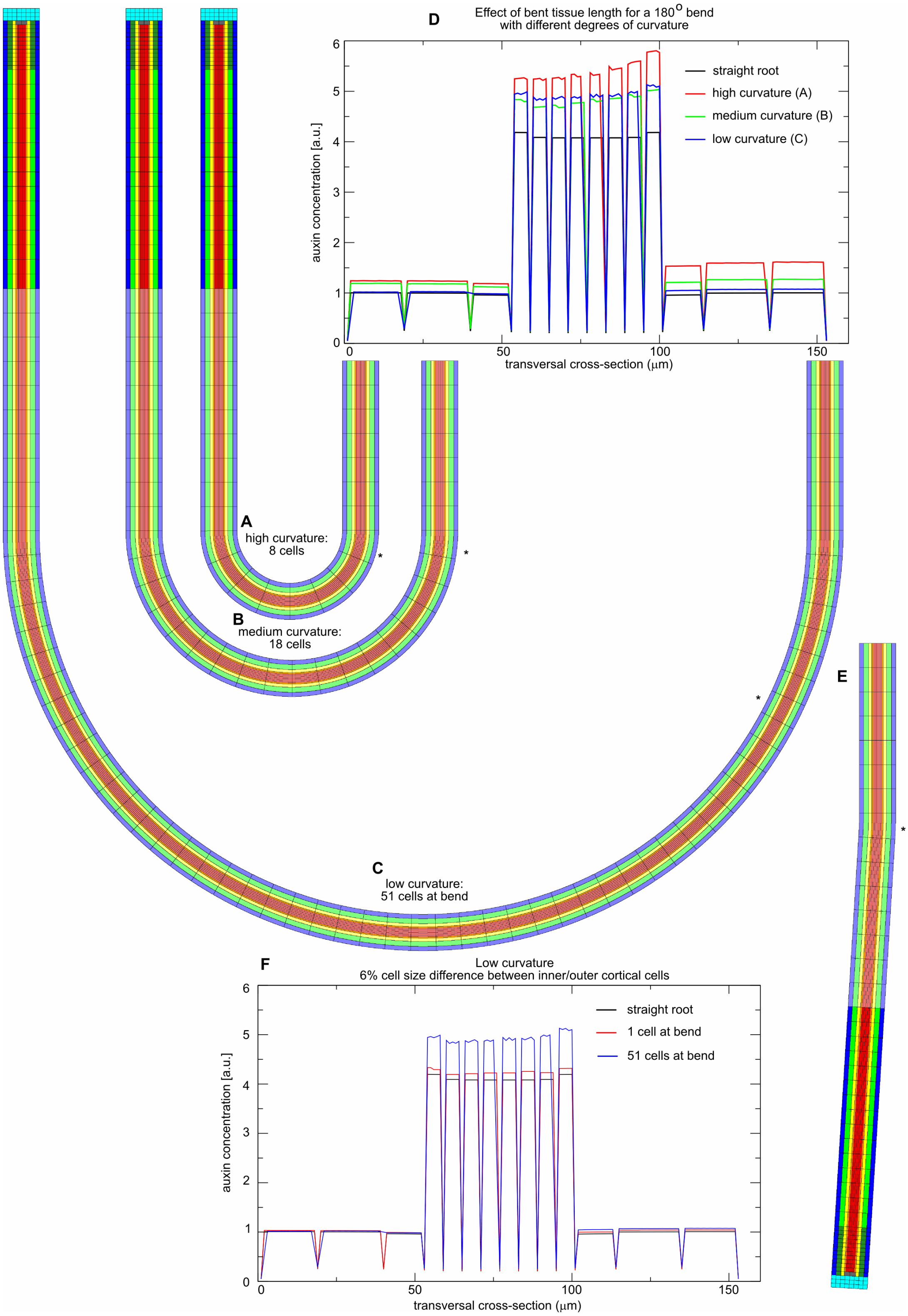
D
Effect of bent tissue length for a 180° bend
with different degrees of curvature
auxin concentration [a.u.]
transversal cross-section (μm)
straight root
high curvature (A)
medium curvature (B)
low curvature (C)
A
high curvature:
8 cells
B
medium curvature:
18 cells
C
low curvature:
51 cells at bend
E
F
Low curvature
6% cell size difference between inner/outer cortical cells
auxin concentration [a.u.]
transversal cross-section (μm)
straight root
1 cell at bend
51 cells at bend

Figure S11

Figure S11: The magnitude of auxin accumulation depends on the geometry of larger root sections.
Here we explore the effect of larger sections of curved root on the formation of an auxin bias. In the first experiment, we created a 180° bend in an *in silico* root using either a few strongly curved cells or many weakly curved cells. The root is bent 180° over 8 cells **(A)**; 18 cells **(B)**; or 51 cells **(C)**. In our model, given that the distance between the centers of the inner and outer cortex cells is 95 μm, the cell length differences between the inner and outer cortex cells are 38% for the high curvature (A), 17% for the medium curvature (B), and only 6% for the low curvature (C). **(D)** Transversal cross-sections through the center of the cell row indicated by a * in each figure. As expected by cell-size considerations, the increase in auxin at bend is largest for the root with the sharpest bend, the one with the largest cell size differences between the inner and outer sides of the curve. Surprisingly, the model root with the lowest curvatures shows a greater accumulation of the auxin in the vasculature than the root with the medium curvature does. This can not be explained by differences in cell size and thus indicates that the extent of the bent region is also important. **(E)** Model root layout in which a 6% difference in cortical cell size difference is applied to a single row of cells. **(F)** Transversal cross-section comparing auxin levels in (C) and (E), bends that have precisely the same low curvature, and hence cell shape, but differ in length. This comparison shows that only a sufficiently long bend is able to amplify the effect of such a small cell-size difference, due to the lateral fluxes (between vascular and external cell files) that occur over the whole extension of the bend (see S2 and S12 for more about refluxes). These results support the experimental finding that weakly meandering roots, in which the curvature is small but extends through many cells, still consistently form lateral roots with the typical outer bias.

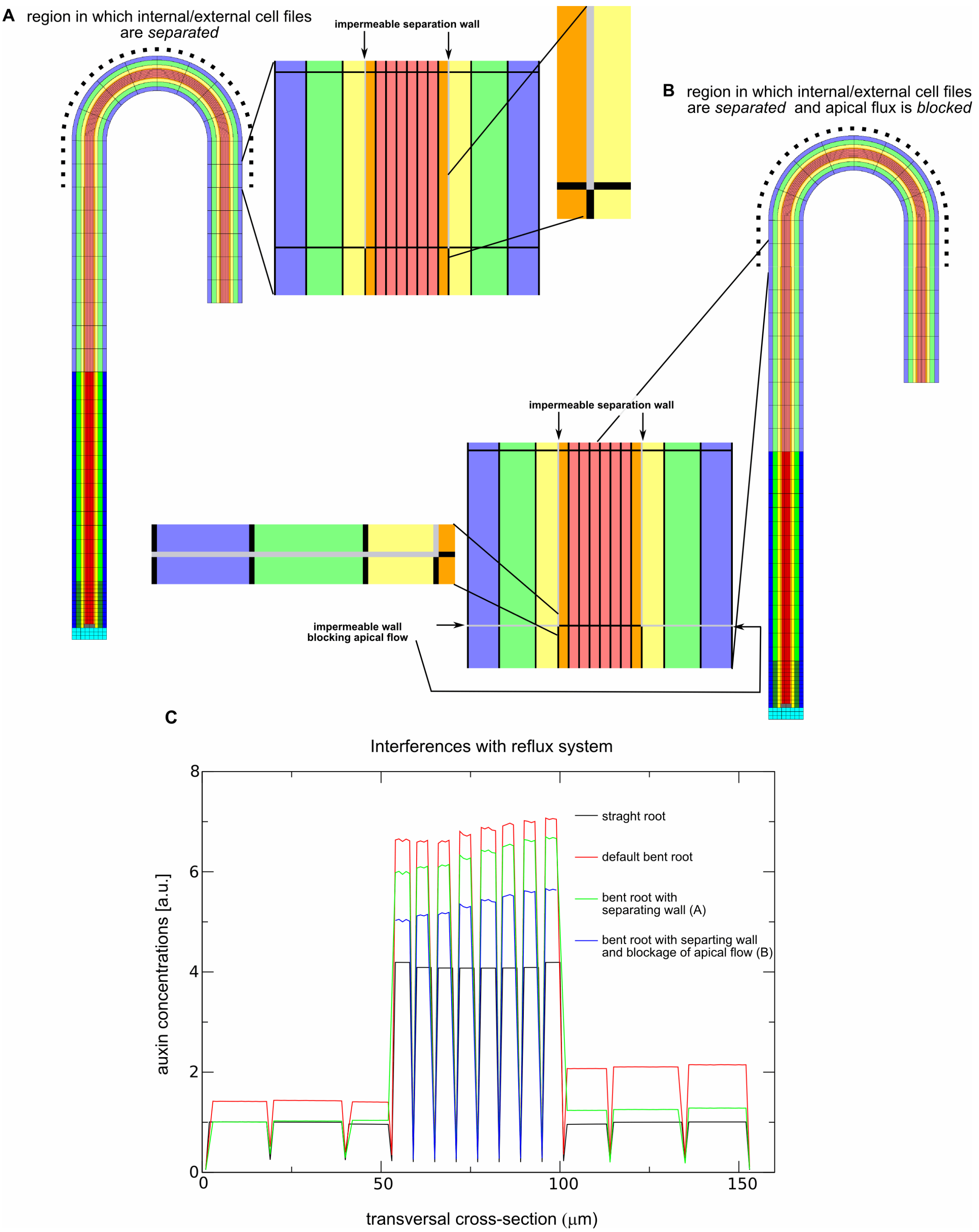
A region in which internal/external cell files are *separated*
impermeable separation wall
B region in which internal/external cell files are *separated* and apical flux is *blocked*
impermeable separation wall
impermeable wall blocking apical flow
C
Interferences with reflux system
straght root
default bent root
bent root with separating wall (A)
bent root with separting wall and blockage of apical flow (B)
auxin concentrations [a.u.]
transversal cross-section (μm)

Figure S12

Figure S12: Blocking communication between vascular and external tissue: the role of pure cell length and reflux.
To obtain a better estimate on the relative contributions of cell length changes and reflux loops to rising auxin levels and pericycle auxin maximum formation on the outside of a bent root, we did simulations in which the reflux loops occurring between the vascular and the flanking external cell files were blocked by means of introducing auxin-impermeable layers. **(A)** Close-up of the *in silico* root layout that now includes a vertical barrier preventing auxin from moving between the pericycle and the endodermal cell files. This blockage is applied to the region of the bend, indicated by the dotted line. **(B)** To further reduce reflux, we modified the layout of (A) by further adding horizontal, auxin-impermeable barriers to the external cell file walls at the boundaries between the bent region and the unbent regions. **(C)** The resultant steady state auxin concentrations at the bend are shown in comparison to a straight root (black) and a bent root without artificial transport barriers (red). When auxin transport between the vascular and external cells is separated, as in (A), the auxin increase in the external cell files on the inside of the bend disappears. However, due to the cell length differences, as well as a reflux loop spanning the whole bent region, an increase in auxin concentration along the outer regions can still be observed, although its magnitude is much reduced (green). Thus, blocking a direct exchange between the vascular and external cell files has a strong effect on the auxin increase and bias. A much more dramatic decrease in the amount of auxin accumulation is observed in case (B), in which the formation of reflux is further blocked (blue). (Note, that in all three cases the net downward auxin flux through the root is exactly the same. Also, lateral auxin transfer is still occurring within the vascular tissue itself, which is why the inner pericycle cells present a slight auxin increase). Thus, we here show that the reflux loop generated due to bending is important for the magnitude of auxin accumulation in the vasculature, as well as for the significant increase of auxin in the external cell files. When we simulate the AUX1-auxin feedback while using layout (B), the amplified pericycle auxin maximum fails to form (data not shown). This reveals that – in the absence of the reflux loop, and given the same parameter setting for the AUX1-auxin feedback – cell shape changes alone are not sufficient to trigger the large auxin increase in the pericycle cells. Note that within a restricted parameter space it would still be possible to trigger the AUX1-auxin amplification. In such a situation, however, the system would become highly sensitive. Any perturbation would be picked up, and as a consequence the dynamics would loose robustness.

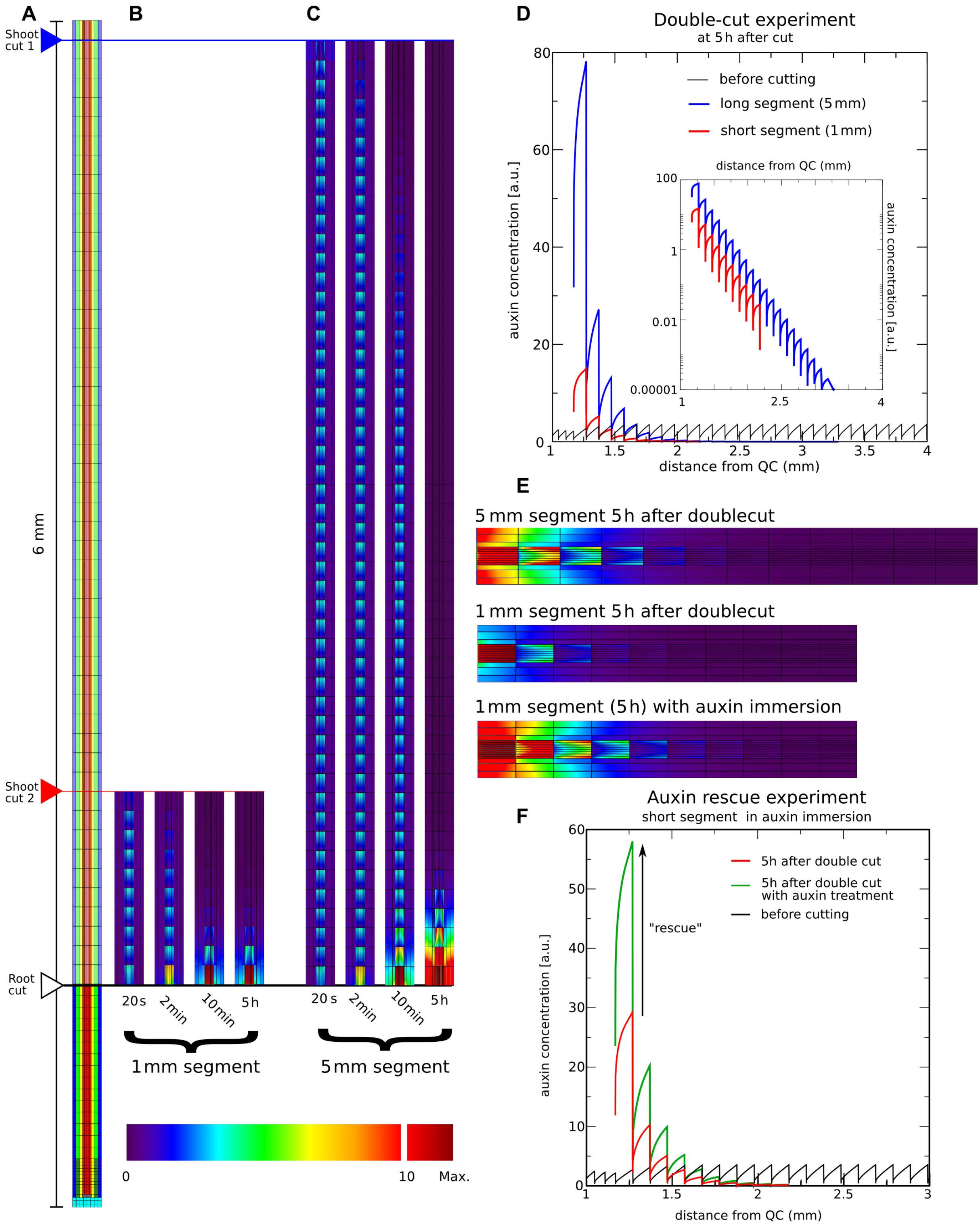

A
B
C
D
Shoot cut 1
Shoot cut 2
Root cut
6 mm
20 s
2 min
10 min
5 h
1 mm segment
5 mm segment
0
10
Max.
Double-cut experiment
at 5 h after cut
before cutting
long segment (5 mm)
short segment (1 mm)
distance from QC (mm)
auxin concentration [a.u.]
E
5 mm segment 5 h after doublecut
1 mm segment 5 h after doublecut
1 mm segment (5 h) with auxin immersion
F
Auxin rescue experiment
short segment in auxin immersion
5h after double cut
5h after double cut with auxin treatment
before cutting
"rescue"

Figure S13

Figure S13: Modeling the effect of cutting roots.
Our model confirms classical experiments that demonstrated a role for shoot-derived auxin in lateral root initiation. Classical work from Bonnett and Torrey [8] revealed that after cutting away the root tip of Field Bindweed (*Convolvulus arvensis*), lateral roots form just proximal to the location of the cut. When whole segments of the root were cut out (no root tip nor shoot), segments still formed laterals at the distal end, as long as they were sufficiently long (>1.5 mm). Smaller segments lacked the capacity to form lateral roots. This capacity could be restored by immersing the segment in auxin. When simulating the interruption of auxin fluxes to and from the root-tip in our default model, i.e. the removal of both the root tip and the shoot, we observe auxin accumulation at the basal end of the segment, with total accumulated amounts depending on the segment size (A–C). Immersing the *in silico* root segment in auxin [2] provokes increases in the accumulation levels. Thus, the physiological experiments and the model are in agreement, further supporting the hypothesis that auxin levels in the vasculature cause the initial divisions required for lateral root formation.

(A) Intact *in silico* root segment, which, by appropriate boundary conditions, is proximally receiving influx from the shoot. The total length described is $\approx$6 mm. The intact root undergoes a simultaneous removal of both root and shoot. The position of the distal cut is indicated by 'root cut'. Root cutting is implemented by altering the boundary conditions such that no transport occurs over the cross-section. Shoot cuts, which are performed by interrupting the auxin influx, are implemented at two different locations, forming root segments of either 1 mm or 5 mm. **(B,C)** Dynamics of auxin pattern in short (B) and long (C) root segments after root and shoot removal. Note that the longer segment distally accumulates much higher auxin concentrations within the same time interval. **(D)** Auxin concentration profiles along the pericycle cell file, indicating larger concentrations at the distal end of the longer segment, compared to the shorter one. inset: log-linear plot of auxin concentrations with distance from QC, in which the exponential slop of the auxin gradient becomes apparent. **(E)** Auxin rescue experiment, *in silico*. Top: the distal end of the 5 mm incised root segment. Middle: auxin profile of the 1 mm root segment. Bottom: auxin profile in the 1 mm root segment, when incision is combined with immersion of the segment in auxin. Immersion is modeled by allowing for a peripheral influx of auxin from all sides of the root segment. Note that the auxin accumulation pattern of the 1 mm root segment resembles, after 5 h auxin treatment, the pattern observed in the tip of the 5 mm segment without the auxin treatment. **(F)** Graph showing an increase in auxin concentration in pericycle cell files due to auxin treatment.

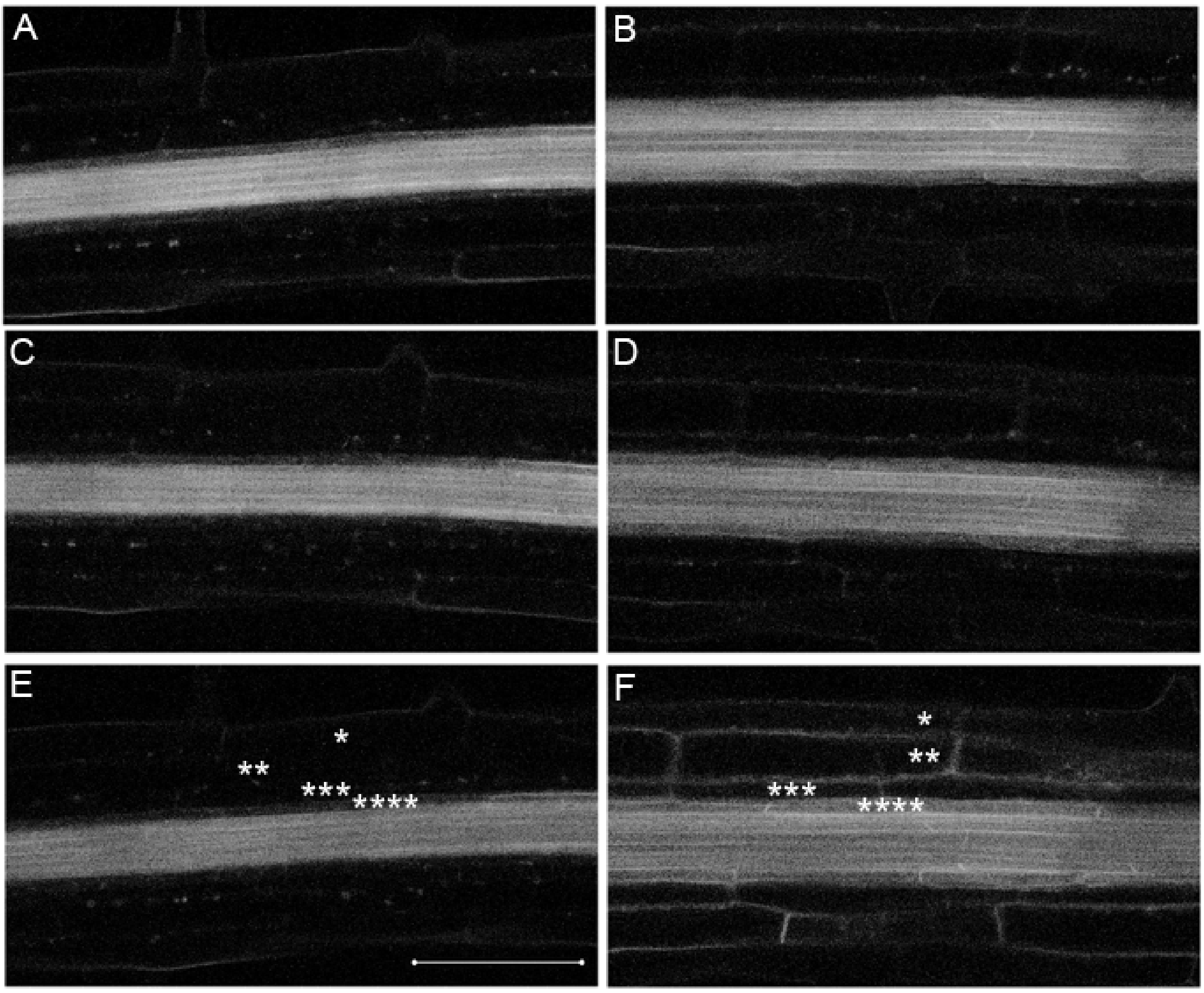

Figure S14: AUX1 is auxin responsive.
(A,C,E) water treated AUX1:YFP reporter lines show no alteration in AUX1 membrane localization outside of the stele. **(B,D,F)** Ectopic membrane localized AUX1:YFP outside the stele is absent at (B) 30 min; present within (D) 120 min; and apparent within (F) 180 min of 10 μM IAA application. Panels (A,B) 30; (C,D) 120; and (E,F) 180 min after treatment. Epidermal*, cortical**, endodermal***, and pericycle**** cell files are labeled. Scale bar: 100 μm.

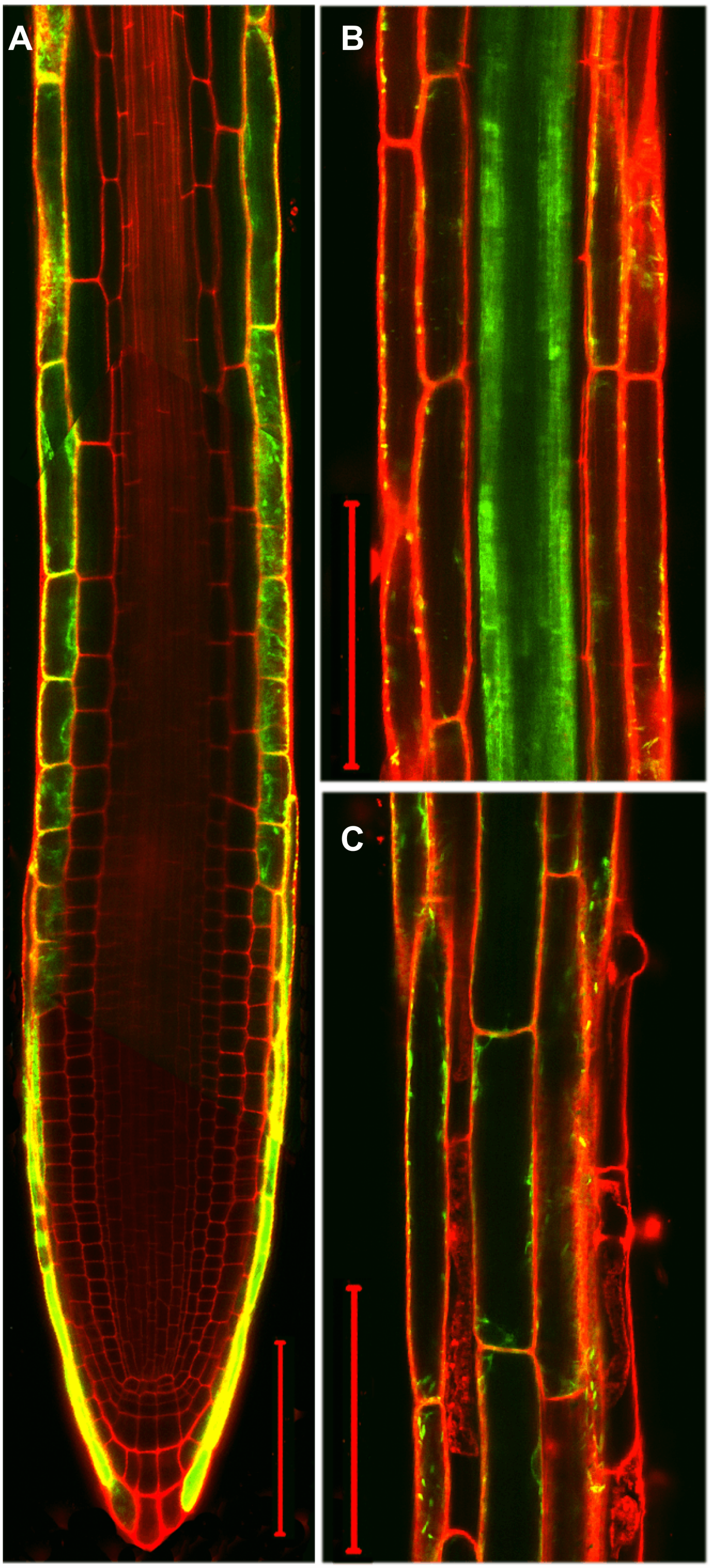

Figure S15: J0951 induces pericycle expression in the DZ.
(A) In a transactivation system, J0951:GAL4 drives UAS:GFP expression in the root cap and epidermal cell files in tissues between the root tip and distal DZ. **(B)** In mature root tissue J0951 is specific for pericycle cells. **(C)** Epidermal expression is reduced in the late DZ. Scale bars, 100 μm.

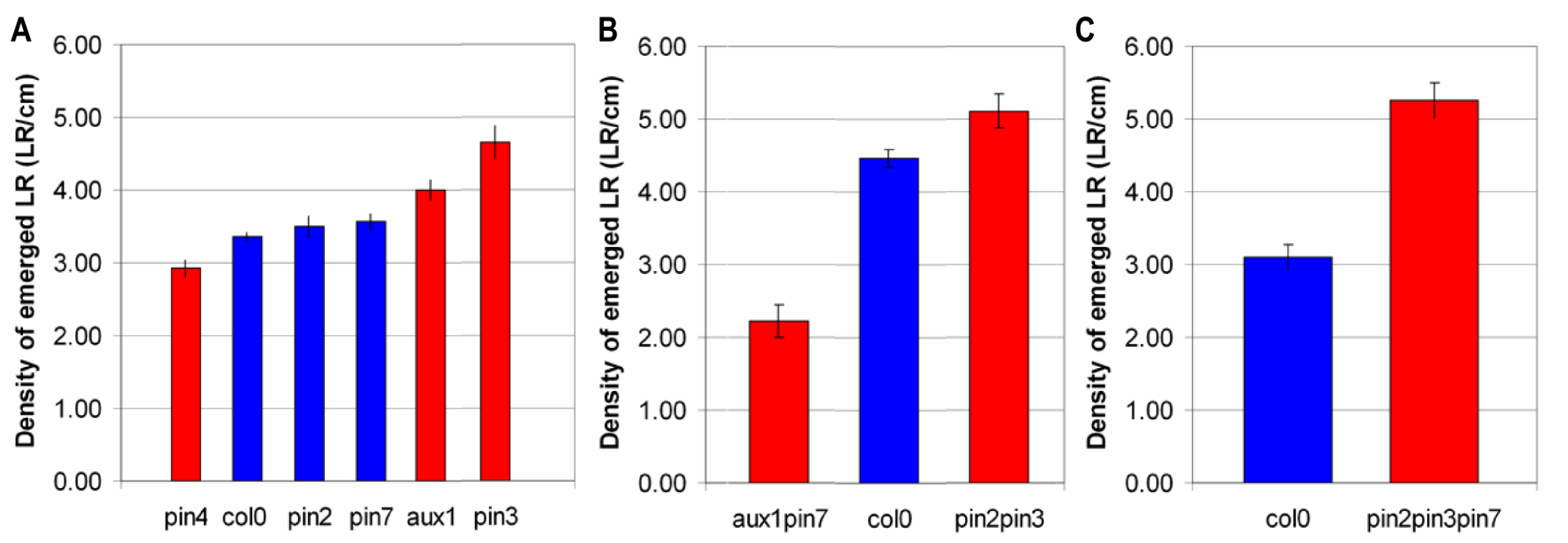

Figure S16: Auxin transport proteins regulate lateral root densities.
Lateral root density (emerged lateral roots/cm) of vertically grown 12 dpg plants for **(A)** single *pin* and *aux1* mutants; **(B)** *pin* and *aux1* mutant combinations; and **(C)** *pin2,3,7* triple mutant. Red bars represent plants with lateral root densities that are significantly different from wildtype based on a Student's t-test ($p<0.05$). Error bars=SEM.

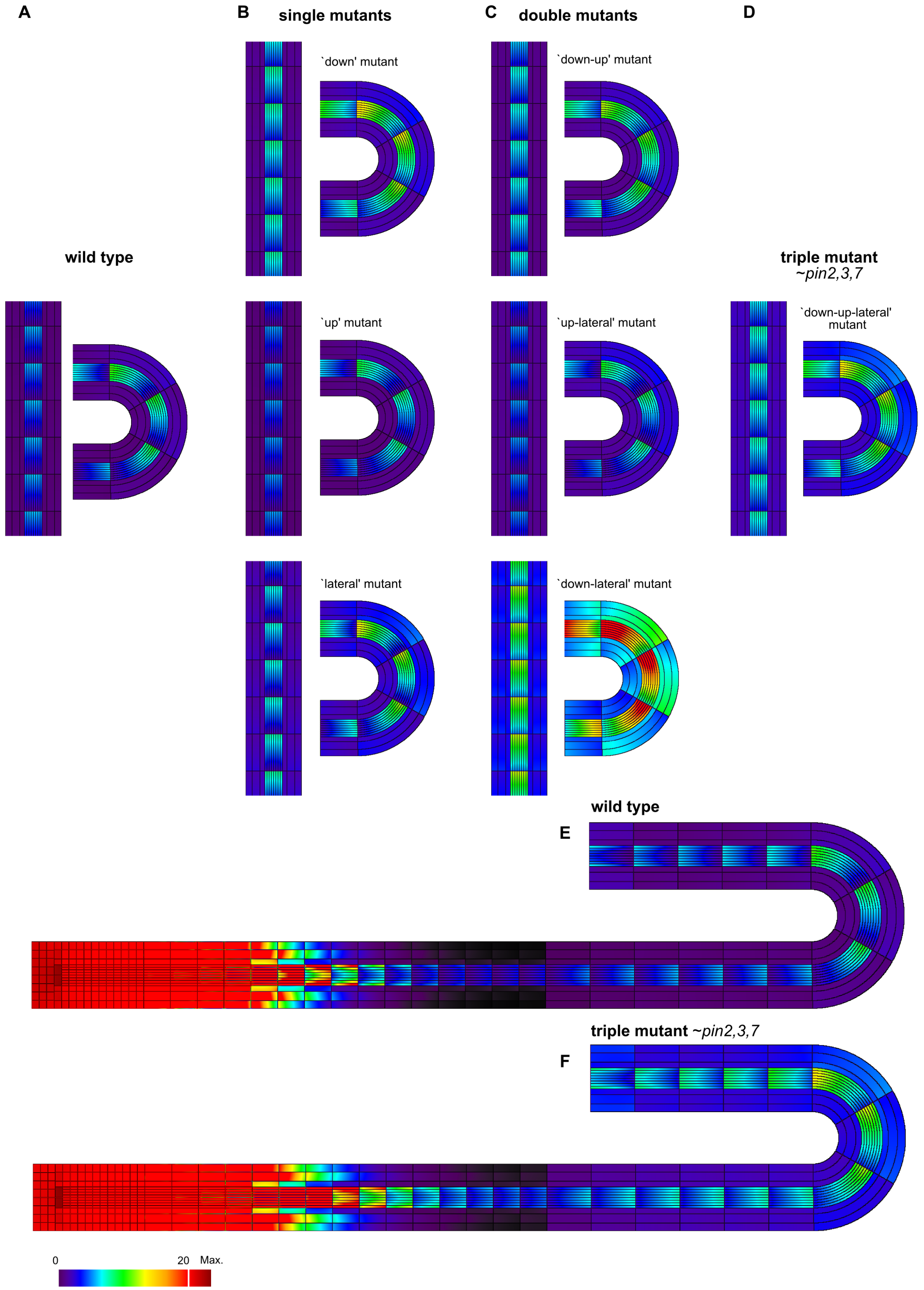
A
B single mutants
C double mutants
D
`down' mutant
`down-up' mutant
wild type
triple mutant
~pin2,3,7
`up' mutant
`up-lateral' mutant
`down-up-lateral' mutant
`lateral' mutant
`down-lateral' mutant
wild type
E
triple mutant ~pin2,3,7
F
0
20
Max.

Figure S17

Figure S17: Dissecting PIN function by simulations of mutants with lateral, apical and basal PIN alterations.
Here, we explore the effect of individually altering the apical, basal and lateral components of auxin permeability. The ‘up mutant’ has reduced apical PIN expression; the ‘down mutant’ has reduced basal PIN expression; the ‘lateral mutant’ has reduced lateral PIN expression. Auxin profiles of DZ segments before and after bending of **(A)** wildtype; **(B)** single mutants; **(C)** double mutants; and **(D)** triple mutant simulations. Full length simulated root shown for wildtype **(E)** and triple mutant **(F)**. The up-mutant shows a decreased tendency to accumulate auxin on the outer side of the curve, while both down- and lateral- mutants present an increased auxin bias at the bend. Double mutants are combinations of the previous single mutants. A striking enhancement of the auxin response to curving occurs for the ‘down-lateral’ mutant. The second most sensitive mutant towards curvature is the triple combination in which PIN expression is decreased in all directions (down, lateral, up), as might be predicted for *pin2,3,7*. PIN expression was reduced by changing $P_{e_{SPIN}}$ from 20 to 5 μm/s and $P_{e_{WPIN}}$ from 5 to 2 μm/s for all cells except the columella cells, which were unaltered. At the same time, the boundary condition at the shoot end was modified such that the net downward auxin flux remained constant for all simulations. To simulate the mutants, apical, basal, and/or lateral PIN expression has been modified in the same way for each cell type, expect for the PIN expression in the columella cells, which was kept the same in all simulations.

Supporting Tables

Table S4: Cortical cell measurements.
Cortical cell measurements for roots that are curved due to graviresponse or manually curved (J-hooked). The radius of the curve is measured at the location of the measured cells.

Location	Measure (μm)	Std Dev	SEM	Ratio
	**Length** :			
Outer cells	184 (n=21)	50.4	11.0	
Inner cells	148 (n=21)	27.0	5.9	
				1.25
	**Width:**			
Outer cells	18.6	2.8	0.6	
Inner cells	18.4	2.6	0.6	
				1.01
	**Radius:**			
Outer cells	775	199	NA	
Inner cells	704	195	NA	
				1.10

Location	Measure (μm)	Std Dev	SEM	Ratio
	**Length:**			
Outer Cells	179 (n=44)	40.8	6.2	
Inner Cells	169 (n=45)	28.3	4.2	
				1.06
	**Width:**			
Outer Cells	17.7	1.9	0.3	
Inner Cells	17.9	1.9	0.3	
				0.98
	**Radius:**			
Outer Cells	952	348	NA	
Inner Cells	883	348	NA	
				1.08

Supporting Movies

Movie S1:

Increased auxin response precedes asymmetric division of the founder pericycle cells.
DR5:vYFP root was gravitropically stimulated for 4 h, then placed horizontally and imaged every 10 min thereafter. Arrow indicates approximate height at which an endodermal cell shows increased auxin response prior to the strong increase in founder cells. Root oriented such that the top of the frame is closest to the shoot, bottom to the root tip.

Movie S2:

AUX1:YFP levels increase in pericycle cells on the outside of a curve prior to asymmetric cell division.
Root was gravitropically stimulated for 4 h prior to imaging; frames taken 10 min apart. Root oriented such that the top of the frame is closest to the shoot, bottom to the root tip.

Movie S3:

Simulation of AUX1 and auxin feedback, showing regulatory dynamics and auxin changes after root bending.
Left panel, AUX1 expression levels indicated by color bar above. Right panel, auxin concentration levels, indicated through heatmap color bar above. Scale bar: 100 μm. Time after root bending is indicated.

Movie S4:

Control simulation of AUX1 and auxin feedback within a straight root, confirming stable regulatory dynamics in the absence of curvature.
Upper panel, AUX1 expression levels indicated by left color bar. Lower panel, auxin concentrations indicated by heatmap on the right. Scale bar: 100 μm. Simulation is started with a root devoid of auxin. Time after root bending is indicated.

Movie S5:

Simulation of AUX1 and auxin feedback, showing regulatory dynamics and auxin changes after root bending over the whole extension of the root.
Down stream effects due to root curvature can be observed, that travel through the root in the distal direction. Left panel, AUX1 expression levels indicated by color bar above. Right panel, auxin concentration levels, indicated through heatmap color bar above. Scale bar: 100 μm. Time after root bending is indicated.

Movie S6:

PIN7:GFP marker line showing a decrease in fluorescence in a region of the vasculature preceding the formation of a single lateral root primordium.
Lateral root primordium forms on the shoot side of the region with decreased fluorescence (indicated by arrow); root is oriented such that the top of the frame is closest to the root tip. Root was gravitropically stimulated for 4 h prior to imaging; frames taken 10 min apart. Punctate fluorescence that accumulates over time, here seen especially in the cytoplasm of cortical and endodermal cells, was also observed when untransformed roots were imaged this way, and thus is not part of the GFP signal.

References

1. Peaceman DW, Rachford HH Jr (1955) The numerical solution of parabolic and elliptic differential equations. J Soc Indust Appl Math 3:28–41.
2. Grieneisen VA, Xu J, Marée AFM, Hogeweg P, Scheres B (2007) Auxin transport is sufficient to generate a maximum and gradient guiding root growth. Nature 449:1008–1013.
3. Kramer EM, Frazer NL, Baskin TI (2007) Measurement of diffusion within the cell wall in living roots of *Arabidopsis thaliana*. J Exp Bot 58:3005–3015.
4. Delbarre A, Muller P, Guern J (1998) Short-lived and phosphorylated proteins contribute to carrier-mediated efflux, but not to influx, of auxin in suspension-cultured tobacco cells. Plant Physiol 116:833–844.
5. Swarup R, Kramer EM, Perry P, Knox K, Leyser HM, et al. (2005) Root gravitropism requires lateral root cap and epidermal cells for transport and response to a mobile auxin signal. Nat Cell Biol 7:1057–1065.
6. Kramer EM (2004) PIN and AUX/LAX proteins: their role in auxin accumulation. Trends Plant Sci 9:578–582.
7. Kramer EM (2008) Computer models of auxin transport: a review and commentary. J Exp Bot 59:45–53.
8. Bonnett HT Jr, Torrey JG (1965) Chemical control of organ formation in root segments of Convolvulus cultured in vitro. Plant Physiol 40:1228–1236.
